# One step laser-induced synthesis of a bimetallic iron–cobalt sulfide for efficient solar light driven, Fenton-like and electrochemical catalysis†

**DOI:** 10.1039/d5ra03059e

**Published:** 2025-07-23

**Authors:** Tomáš Křenek, Lukáš Vála, Palaniappan Subramanian, Saleem Ayaz Khan, Ján Minár, Martin Koštejn, Rostislav Medlín, Petr Mikysek, Věra Jandová, Veronika Vavruňková

**Affiliations:** a Faculty of Mechanical Engineering, Department of Material and Engineering Metallurgy, University of West Bohemia Univerzitní 8 301 00 Pilsen Czech Republic tkrenek@ntc.zcu.cz valal@fst.zcu.cz; b New Technologies-Research Center, University of West Bohemia Univerzitní 8 306 14 Pilsen Czech Republic; c Institute of Chemical Process Fundamentals, Czech Academy of Sciences Rozvojová 135 165 02 Prague 6 Czech Republic; d Institute of Geology, Czech Academy of Sciences Rozvojová 269 165 00 Praha 6 Czech Republic

## Abstract

Pulsed laser irradiation of an equimolar mixture of FeS_2_ and CoS_2_ onto a Ta substrate results in the one-step formation of bimetallic iron cobalt sulfide. The use of complementary analytical techniques, such as scanning electron microscopy, Raman spectroscopy, X-ray photoelectron spectroscopy, X-ray diffraction, high-resolution electron microscopy, and electron diffraction, confirmed the presence of nanocrystalline cobaltpentlandite [FeCo_8_S_8_] and maghemite [γ-Fe_2_O_3_]. The mechanism by which this occurs involves the reactive interaction of laser-ionized Fe, Co, and S species, which subsequently undergo rapid non-equilibrium cooling and deposition. The higher deposition tendency of CoS_2_ along with iron ions/atoms leads to the formation of FeCo_8_S_8_. This proposed mechanism is supported by density functional theory (DFT), which provides a deeper understanding of the higher thermodynamic stability of Fe in Co_1−*x*_Fe_*x*_S_2_ compared with Co in Fe_1−*x*_Co_*x*_S_2_. The FeCo_8_S_8_-based deposit exhibited enhanced catalytic efficiency for methylene blue daylight-driven and Fenton-like degradation. In contrast, for solar light-driven degradation of sulfamethoxazole and trimethoprim, the FeCo_8_S_8_-based deposit does not show enhanced catalytic activity compared to FeS_2_ and CoS_2_. Additionally, electrochemical testing of the oxygen evolution reaction (OER) revealed significantly improved performance for the FeCo_8_S_8_-based deposit compared to FeS_2_ and CoS_2_ individually.

## Abstract 2

Synthesis of a multifunctional bimetallic iron–cobalt sulfide with enhanced catalytic and electrochemical performance was achieved using one-step pulsed laser deposition.

## Graphical abstract

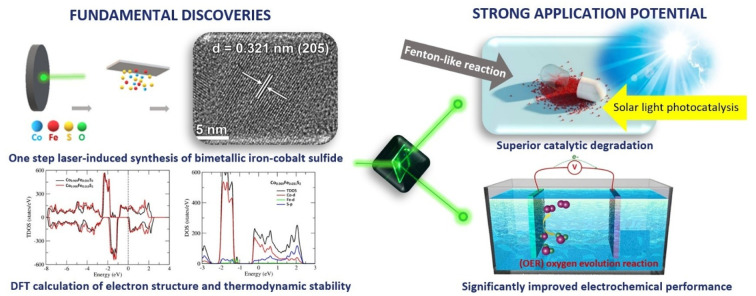

## 1. Introduction

Currently, nanocrystalline metal sulfides attract great scientific interest due to their tunable optical, physical and chemical properties. Metal sulfides represent semiconducting compounds where the metal cations can be included in mono-, bi- or multi-form.^1^ Metal monosulfides have received attention over the past few decades owing to their suitable electronic band gap, band position, exposed active sites, and promising catalytic and photocatalytic activities. Compared to metal oxides, metal sulfides in many cases exhibit shallow valence bands^2,3^ and a smaller effective mass, which allows a strong quantum size effect.^4,5^ Metal sulfides can be prepared with various sizes, morphologies, and chemical and phase compositions, which allow excellent tunability of the photon response over a broad range of the solar spectrum. Metal sulfides are also used in rechargeable batteries, specifically as electrocatalytic materials suitable for the oxygen evolution reaction (OER) and oxygen reduction reaction (ORR).^6^ In this field, extensive efforts have been devoted to replacing noble metals with various non-noble materials such as metals,^7^ metal oxides,^8^ metal chalcogenides.^9^ However, the use of these materials as electrode components is still limited by the weak synergy between intrinsic activity, selectivity, and electrical conductivity.^10^ Metal oxides exhibit good intrinsic catalytic activity; however, their electrical conductivity is not sufficient.^11^ Metal sulfides exhibit higher conductivity and similar intrinsic activity compared to metal oxides,^12^ which is caused by the electronegativity and atomic radius differences between oxygen and sulfur atoms. The properties of monometallic sulfides may be further exceeded by those of bimetallic sulfides because of the synergistic effect between metal sites and also due to the changes in bond distance, bond energies, and bond modes.^13,14^ Thus, bimetallic sulfides offer enhanced characteristics such as optimal band gap, good electronic conductivity, and intrinsic activity, which are desirable for photo- and electrochemical catalysis. In comparison to the monometal sulfides, bimetal sulfides display rich redox chemistry due to the participation of two metals in addition to higher electronic conductivity than their monometal sulfide and oxide counterparts. Consequently, a significant enhancement of the electrochemical redox properties and synergistic effects from two metal ions are reported for bimetal sulfides applied in lithium ion battery electrodes, sodium ion battery electrodes, metal–air battery electrodes, supercapacitors and water splitting devices.^14–16^ Despite the exceptional application potential of bimetallic sulfides, the phase and structural design of this compound represents a challenge.^17^ The formation of bimetallic sulfides is more difficult than that of their monometallic counterparts because the crystallization of bimetallic sulfides is strongly influenced by two metal element properties with distinct kinetics and thermodynamic parameters.^18^ Specifically, bimetallic iron–cobalt sulfide has been shown to exhibit excellent photocatalytic activity (for the photodegradation of methylene green and rhodamine-B)^19^ and, electrochemical performance in terms of OER activity,^20–24^ which are enhanced compared to monometallic sulfides. Hierarchical cobalt–iron sulfide anchored on carbon fibers provides practical support for efficient capture of Hg^0^ and outperforms traditional iron sulfide and cobalt monosulfide.^25^

Up to now, bimetallic iron–cobalt sulfides, have been synthesized by several steps containing hydrothermal or solvothermal routes: FeCo_8_S_8_ and FeCoS_2_ nanosheets on reduced graphene oxide has been prepared using cobalt(ii) acetylacetonate, iron(III) 2,4-pentanedionate, oleylamine, 1-octadecene, oleic acid, and 1-octanethiol precursors;^21^ Fe-doped CoS nanoplate was prepared *via* solvothermal reaction with cobalt chloride hexahydrate, ammonium iron(ii) sulfate, thioacetamide, triethylenetetramine;^20^ Co_*x*_Fe_1−*x*_S_2_ with different Co/Fe atomic ratios was prepared with cobalt nitrate hexahydrate, iron nitrate nonahydrate hydrothermal process and following chemical vapor deposition (CVD) sulfurization treatment;^22^ iron cobalt transition-metal sulfide (FeCoS) on Ni foam with nanosheet arrays was fabricated *via* electrodeposition of cobalt nitrate hexahydrate, iron chloride hexahydrate, thiourea solution;^23^ hollow FeCo_2_S_4_ nanotube multi-tripod arrays supported onto carbon cloth was synthetized by hydrothermal procedure of iron nitrate nonahydrate and cobalt nitride hexahydrate followed by sulfidation process using sodium sulfide nonahydrate;^24^ cobalt–iron sulfide anchored on carbon fibers was prepared using hydrothermal and calcination route with iron nitrate nonahydrate and cobalt chloride hexahydrate and thioacetamide as a sulfur source;^25^ cobalt–iron sulfide has been formed also from furyl and ferrocenyl based dithiocarbamate ligands.^19^ Clearly, the current hydrothermal or solvothermal methods for synthesizing iron–cobalt sulfides typically require the use of challenging, less commonly employed, and potentially toxic chemical compounds, as well as a multi-step process that is both time- and energy-consuming. Furthermore, the reported preparation method often involves drying under specific atmospheric conditions, such as inert gas or vacuum, and may even require additional calcination. Our previous investigations, which were conducted systematically, focused on the pulsed laser deposition of individual monometallic iron sulfide^26–28^ and cobalt sulfide.^29^ These studies have shown that when these materials are deposited on an unheated copper substrate, reactive deposition occurs, leading to the formation of bimetallic compounds such as Cu_5_FeS_4_ and Co_2_CuS_4_. However, when these compounds are deposited onto a tantalum substrate, the deposition of the original FeS_2_ and CoS_2_ phases is favored.

In this contribution, we report on the pulsed laser ablation of equimolar mixed FeS_2_ and CoS_2_ targets, followed by deposition onto a Ta substrate, resulting in the formation of nanostructured FeCo_8_S_8_ and γ-Fe_2_O_3_ phases. This represents a facile one-step process for the formation of bimetallic sulfides. The experimental data were supported by density functional theory (DFT) calculation to provide a deeper insight into the structural stability and electronic structure of the iron–cobalt sulfide system. To demonstrate the multifunctional catalytic properties of the FeCo_8_Fe_8_ phase, the deposit was tested for daylight-driven and Fenton-like catalytic degradation of methylene blue (MB). Additionally, the ability of the FeCo_8_S_8_-based film to decompose sulfamethoxazole and trimethoprim under simulated solar light exposure conditions was investigated. Furthermore, the electrocatalytic activity towards oxygen evolution reaction (OER) were assessed. Comparative evaluations were made of the photo-, Fenton-like, and electrocatalytic characteristics of the FeCo_8_S_8_-based coating with those of individual monometallic FeS_2_ and CoS_2_.

## 2. Experimental and computational methods

### 2.1 Laser-induced synthesis

#### 2.1.1 Preparation of target

The FeS_2_–CoS_2_ pellet were prepared at 100 atm on a hydraulic press from equimolar mixture of commercially available iron disulfide (FeS_2_, 99.98%, Aldrich) and cobalt disulfide (CoS_2_, 99.98%, Aldrich) powders, which were homogenized by mixing in agate bowl under ambient air before pressing. Individual FeS_2_ and CoS_2_ pellets were prepared under the same pressing conditions.

#### 2.1.2 Deposition of metal sulfide films

A 3^rd^ harmonic of pulsed Nd:YAG laser with base wavelength 1064 nm (model Q SMART 850) with wavelength 355 nm and energy 180 ± 5 mJ per pulse, pulse duration: 10 ns, with repetition rate 10 Hz, was focused by lens (*f* = 15 cm) on the spot area of 0.02 cm^2^ (laser fluence 2.5 J cm^−2^) was used for ablation of an equimolar mixture of FeS_2_–CoS_2_ target. As a substrate tantalum foil (Ta, 99.98%, Aldrich) was used. The highly focused UV laser (355 nm) irradiation of the FeS_2_–CoS_2_ pellet results in fast deposition (the glassy chamber becomes dark immediately after the first pulses) and leads to the formation of a homogeneous dark adhesive Fe–Co–S deposit on the Ta substrate. The ablation was accompanied by a significant blue luminescent zone filling the entire space of the reactor chamber, which suggests high ionization of Fe–Co–S–O species. A vacuum (2 × 10^−4^ Pa) inside a simple tubular Pyrex reactor (70 mL in volume) was achieved using a turbomolecular pump (HiCube, Pfeiffer). The reactor was equipped with borosilicate glass windows. The duration of irradiation was 6 minutes. The target of the FeS_2_–CoS_2_ pellet with a diameter 10 mm and height of 4 mm was positioned vertically in the center of the reactor, and the substrate was positioned perpendicular to the Ta target. For each sample deposition, the vacuum chamber was first opened and the clean substrate was placed inside the Pyrex chamber. The reactor was then closed, and the pressure was lowered. After irradiation, the pressure was increased to atmospheric pressure, the chamber was opened, and the sample with the resulting films was taken for examination. Coats based on individual monometallic FeS_2_ and CoS_2_ (used as control samples) were deposited under the same conditions.

### 2.2 Physico-chemical characterization

X-ray diffraction analysis (XRD) of the input powder, irradiated pressed target, and deposit was performed using a Bruker D8 Discover diffractometer equipped with a silicon-strip linear LynxEye detector and a focusing germanium primary monochromator of Johansson type providing Cu Kα_1_ radiation (*λ* = 1.54 056 Å). Data for mineral identification were collected in the 2*θ* range of 5–70° with a step size of 0.016° and a counting time of 1 s at each step, and detector angular opening of 2.935° phase identification was performed using Diffrac.Eva software v7.3 and the ICDD PDF-4/Axiom 2023–2026 database (Bruker AXS GmbH, Karlsruhe, Germany; Release 2025). Semi-quantitative estimation of mineral composition was performed using the reference intensity ratio method implemented in the Diffrac.Eva software. A Scanning Electron Microscope (SEM; Tescan Indusem) equipped with a Bruker XFlash® 5010 energy-dispersive X-ray spectrometer (EDS; resolution 125 eV) was used to image the surface morphology and measure the elemental composition of layers. Measurement was carried out with accelerating voltage 15 kV. Raman spectra were obtained using a DXR Raman microscope with a diode-pumped solid-state laser emitting at 532 nm using high-resolution gratings working in the range of 50–1800 cm^−1^ and spectral resolution 2 cm^−1^ FWHM (Full Width Half Maximum). Raman spectra were measured up to 4000 cm^−1^. Surface elemental analyses were performed using a Kratos ESCA 3400 X-ray photoelectron spectrometer (XPS; Manchester, UK). C 1s, O 1s, Co 2p, S 2p, Fe 2p, and Ta 4f lines were observed. For measurement, a small piece of tantalum substrate with a characterized layer was fastened to a carbon tape and mounted onto a holder. All the spectra were corrected by shifting the main carbon C 1s peak to 284.8 eV. An ion gun was used for surface etching (Ar+ ions, 10 mA current, and 1 kV accelerating potential). The Shirley background was subtracted and the elemental compositions of the layers were calculated from the corresponding areas. Transmission electron microscopy (TEM) analysis (particle size and phase analysis) was carried out with a Transmission Electron Microscope JEM 2200FS (Schottky) from JEOL operated at 200 kV with a CCD Gatan (Digital Micrograph software), in-column Omega energy filter 366 for EFTEM and EELS analysis, STEM mode with HAADF detector and EDS 80 mm^2^ SDD (Silicon Drift Detector) X-Max detector from Oxford on scraped samples that were subsequently dispersed in ethanol followed by the application of a drop of diluted suspension on a polymer/carbon coated Cu grid. The diffraction patterns were evaluated using the JCPDS-2 and ProcessDiffraction software package.^30^

### 2.3 Photocatalytic and electrocatalytic measurements

The Fenton-like and solar-light driven catalytic activity of the Fe–Co–S deposit compared with individual FeS_2_ and CoS_2_ deposits was evaluated in terms of MB degradation in aqueous solution in the presence of H_2_O_2_ and in the absence of light (Fenton-like process) and under daylight exposure with an intensity of *Φ* ∼ 700 lx (solar daylight photocatalysis). The MB solution contained 64.2 mmol L^−1^ H_2_O_2_ (H_2_O_2_ was present only in the Fenton-like process) and 0.1428 mmol L^−1^ MB. The total volume of the solution was 3.5 mL. The Ta substrate with the deposit (covered area of 5 × 5 mm) was placed inside the square quartz on the bottom of the reaction cell (base 1 × 1 cm, height 4.5 cm) filled with MB solution, and the depletion of MB was measured every 20 minutes using a DU 730 Beckman Coulter spectrometer. The catalytic activity of the Fe–Co–S deposit was tested also in terms of solar light driven photocatalytic degradation of two selected antibiotics, trimethoprim (TMP) and sulfamethoxazole (SMX). As a source of sun-light, a visible-light sun simulator (100 W, Oriel LCS 100) with an intensity: 100 klx. UV-Vis (ultraviolet-visible) spectra of TMP and SMX depletion were measured using a Shimadzu UV 1800 spectrophotometer with a resolution of 1 nm. UV-Vis spectra were measured for the first 5 h (each hour) and after each 5 hours during the next 24 h. The electrochemical properties were characterized using a Biologic SP-150 electrochemical workstation at room temperature (25 °C). A three-electrode electrochemical flooded cell setup with a graphite rod as the counter electrode and a Hg/HgO electrode (Gaoss Union, Wuhan, China) was used as the reference electrode. CoS_2_/FeS_2_/Fe–Co–S film deposited on tantalum sheet was employed as the working electrode. To evaluate the electrocatalytic water oxidation reaction, the cyclic voltammograms were recorded at 10 mV s^−1^ in the potential range between 0.98 and 1.8 V *versus* Reversible Hydrogen Electrode (RHE) in an Ar-saturated 1.0 M KOH solution. The electrode stability test was carried out with Fe–Co–S deposit on Ta by chronoamperometry at 1.7 V *vs.* RHE in a 1.0 M KOH solution (see Fig. S15†). The EIS measurements were recorded in a frequency range from 100 kHz to 0.1 Hz with an amplitude of 5 mV (peak-to-peak) at open circuit potential.

### 2.4 Crystal structure and DFT calculations

The crystal structures of CoS_2_ and FeS_2_ were obtained from Crystallography Open Database.^31^ For the desired Fe concentration in CoS_2_ we constructed the 2 × 2 × 2 and 3 × 3 × 3 supercells and substituted Fe at Co occupation at different concentrations in Co_1−*x*_Fe_*x*_S_2_ and *vice versa* for Fe_1−*x*_FCo_*x*_S_2_. For the calculation we used the projector-augmented wave method (PAW)^32^ implemented in the Vienna *Ab initio* simulation package (VASP).^33,34^ The Perdew, Burke, and Ernzerhof generalized gradient approximation (PBE-GGA) was used for the exchange-correlation functional.^35^ The calculations are performed in several successive steps. We used the VASP code for geometry optimization, electronic structure, and magnetic calculations. All convergence parameters in the code were carefully checked. In the calculation, the 3d^7^4s^2^ electrons of Co, 3d^6^4s^2^ electrons of Fe, and 3s^2^3p^4^ electrons of S were treated as valence electrons. In VASP code the geometries have been relaxed using the conjugate gradient method with forces estimated using the Hellman–Feynman theorem. The energy cut-off was set to 350 eV. A *Γ*-centered *k*-point mesh of 4 × 4 × 4 was used for the Brillouin zone sampling. The energy and force convergence criteria were set as 10^−6^ eV and 10^−3^ eV Å^−1^, respectively.

## 3. Results and discussion

### 3.1 Spectroscopic characterization of input materials

The XRD pattern of the original CoS_2_ powder (Fig. S1a†) confirms the expected majority of the cubic cattierite [CoS_2_] (PDF 04-003-1962) and trace amount Co–Fe sulfates, *e.g.*, cobaltkieserite [CoSO_4_·H_2_O] (PDF 01-070-2104; 3.39 Å and 2.50 Å) and mikasaite [Fe_2_(SO_4_)_3_] (PDF 00-033-0679; 6.01 Å and 3.00 Å). FeS_2_ original powder in agreement with XRD (Fig. S1b†) consists of a major ratio of pyrite [FeS_2_] (PDF 04-003-1989), and a trace amount of Fe sulfates, *e.g.*, szomolnokite [FeSO_4_·H_2_O] (PDF 04-014-9807; 3.44 Å and 3.10 Å) and ferricopiapite [Fe_4.67_(SO_4_)_6_(OH)_2_·20H_2_O] (PDF 00-029-0714; 9.06–3.58–3.32 Å). Trace phases were determined based on the *d*-spacings, which are given in parentheses. Their accurate determination is prevented by the low concentrations in the samples. XRD pattern of pressed mixed target (Fig. S1c†) exhibits phase composition consisting of pyrite (PDF 01-071-5208), rhomboclase [FeH(SO_4_)_2_·4H_2_O] (PDF 00-027-0245) and cobaltkieserite (PDF 00-015-0701). Smaller diffraction intensity of cattierite (PDF 00-041-1471; 3.19–2.76–2.47–2.26–1.95 Å) was also detected. Qualitatively, no phase transformations occurred during the preparation of the target; however, indicated by the line intensities of the individual phases, the ratio of both iron and cobalt sulfates increased compared to the original majority of the FeS_2_ and CoS_2_ phases in the individual powders. This observation is consistent with the different form of the measured samples. While powder measurement allows irradiation of entire particles volume and detection of subsurface phases, the hydraulically pressed target exhibits a compact surface with a possible higher ordered orientation, providing stronger diffractions of surface sulfates. In addition, hydraulic pressing causes strong friction at the contact surfaces, which could promote the reaction of surface moisture and sulfides and the formation of a thicker surface layer of sulfates. However, subsequent results show that this surface layer of sulfates is probably removed/decomposed during the first pulses of laser irradiation and does not significantly affect the laser-induced reaction between CoS_2_ and FeS_2_.

The X-ray diffraction (XRD) pattern of the target, analyzed before and after laser ablation, exhibited no discernible alterations in phase composition, indicating that no bulk phase transformations occurred in the remaining target material during the processes of pressing and pulsed laser irradiation.

**Fig. 1:**
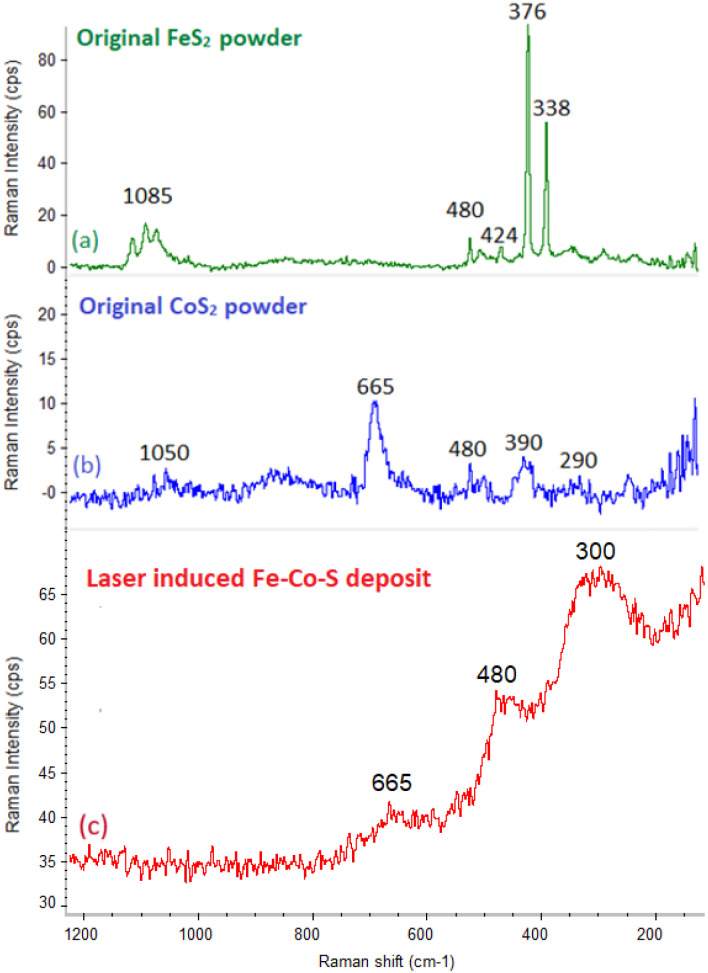
Raman spectroscopy of original FeS_2_ powder (a); original CoS_2_ powder (b); the Fe–Co–S deposit obtained by pulsed laser deposition of mixed FeS_2_–CoS_2_ target (c).

The Raman spectra of the original FeS_2_ powder (Fig. 1a) exhibits intense sharp bands at 376 and 338 cm^−1^ and less intense peaks at 424 and 480 cm^−1^, which corresponds to the pyrite.^37^ The less intense peak centered around 1085 cm^−1^ correspond to the szomolnokite,^37^ which is in agreement with the XRD measurements and it reflects hygroscopic behaviour of the FeS_2_ powder. The CoS_2_ powder shows a typical Raman spectra pattern (Fig. 1b) with a dominant band at 665 cm^−1^ and less intense peaks at 290, 390, 480, 850 and 1050 cm^−1^.^36^ Typically, the peaks at 475, 517, and 676 cm^−1^ are attributed to the CoS phase, while the peaks at 290 and 393 cm^−1^ belongs to CoS_2_,^36^ suggest that the original powder consists of a mixture of both CoS_2_ and CoS phases. The less intense band centered around 1053 cm^−1^ could reflect the minor contribution of the hydrate of CoSO_4_,^37^ which is in line with XRD data and the hygroscopic nature of CoS_2_ powder.

The Raman spectra of the CoS_2_–FeS_2_ target (Fig. S2†) revealed peaks corresponding to the phase compositions of the original FeS_2_ and CoS_2_ powders at 1050, 480, 390, 376, 338, and 290 cm^−1^. Peaks centered at 376, 338 and 424, 480 cm^−1^ correspond to the pyrite, whereas peaks at 290, 390 and 665 cm^−1^ are assignable to CoS_2_.

The appearance of a more intense peak centred at approximately 1050 cm^−1^ in the region of Co/Fe sulfates and intensity decreasing of 665 cm^−1^ peak belonging to CoS are in line with XRD data and are commented above.

### 3.2 Characterization of Fe–Co–S deposit prepared by pulsed laser ablation

#### 3.2.1 Spectroscopic characterization of Fe–Co–S deposit

Typical Raman spectra of the deposit prepared by laser ablation of the mixed CoS_2_–FeS_2_ target (Fig. 1c) shows broad peaks centered at 300, 480, and 663 cm^−1^. This pattern suggests a partially amorphous phase, which could be assigned to both CoS_2_ and FeS_2_ (ref. 36 and 37) species and/or their mixed system, considering that the broad peaks overlap peaks assignable to both individual sulfides. The absence of a peak situated around 1050 cm^−1^ suggests that no significant amount of cobalt or iron sulfates were formed during pulsed laser deposition of the Fe–Co–S coat.

**Fig. 2:**
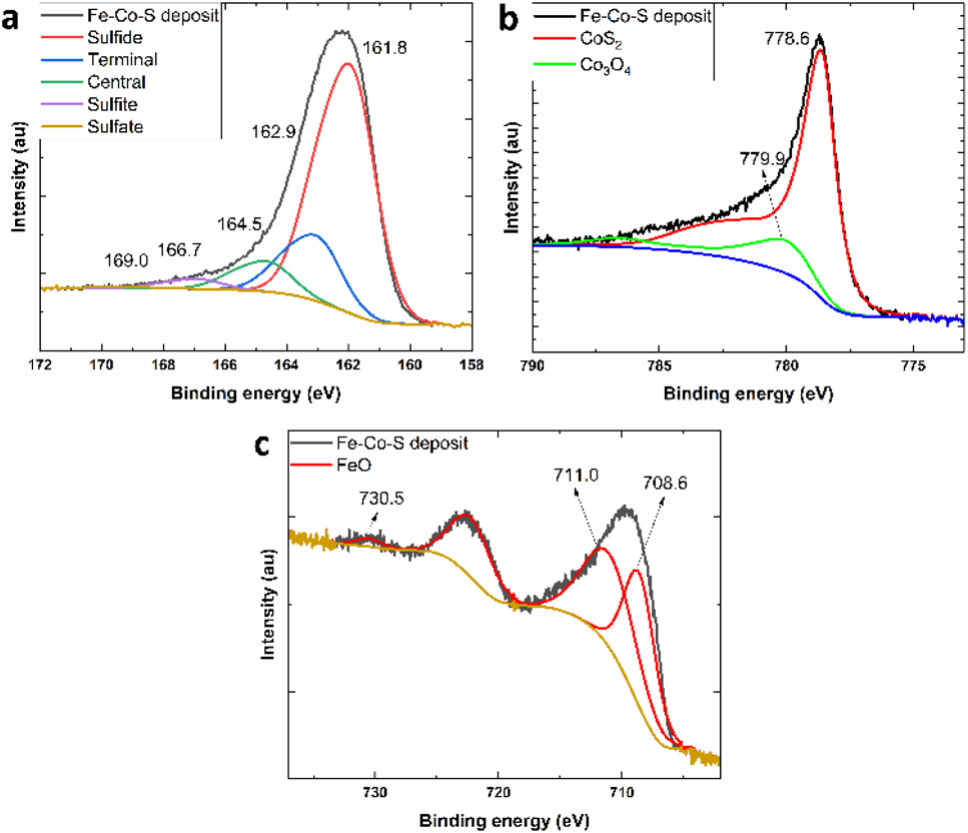
S 2p spectrum (a), Co 2p_3/2_ spectrum (b), Fe 2p spectrum (c) of Fe–Co–S deposit after etching.

X-ray photoelectron spectroscopy measurements were used to determine the surface composition of the Fe–Co–S coat (XPS of the original CoS_2_ powder is provided in Fig. S3 and S4† for comparison with XPS of Fe–Co–S deposit). The XPS quantification results corresponding to the binding energies (BE) of several core levels (C 1s, O 1s, Co 2p, S 2p and Fe 2p) are summarized in Table 1. No Ta signal was detected in any of the Ta 4f spectra of the samples examined. The most significant core levels spectra of the samples after etching are presented in Fig. 2.

**Table 1:** Quantification summary of the XPS data in dependence on etch time

Etch time	Co 2p total [at%]	S 2p sulfate [at%]	S 2p sulfide [at%]	Fe 2p total [at%]	O 1s C=O [at%]	O 1s metal [at%]
0	3.32	3.60	16.61	2.66	23.32	2.11
60	7.23	2.15	26.83	5.14	10.94	4.73
240	12.83	1.04	31.29	8.78	5.11	7.14

The main signal from the C 1s region was ascribed to adventitious carbon, that is, carbon-containing impurities from air. The spectra consisted predominantly of signals ascribed to the C–C bond used for calibration set to 284.8 eV with minor contributions at approximately 287 and 288 eV, which are usually assigned to C–O and C=O, respectively. Spectrum O 1s consisted of broad signal at around 531.7 eV, which can be ascribed to C=O from adventitious carbon and/or surface-bound water or hydroxides (the percentage representation is in the column “O 1s_C=O [at%]” in Table 1). This broad peak might also contain a minor contribution from C–O at approximately 533 eV and 532 eV (only in the surface layer), which is assignable to sulfates. After etching, a peak at approximately 530 eV corresponding to the metal oxide emerged (the percentage representation is in the column “O 1s_metal [at%]” in the Table 1).

The spectra from the S 2p region (Fig. 2a and Table 1 column “S 2p_sulfide [at%]”) showed a broad peak with a tail towards higher binding energies. The S 2p signal is usually fitted by two S 2p_3/2_ and S 2p_1/2_ peaks with a 1.16 eV shift between the peaks. The measured spectra contained several contributions. The main peak centered at 161.8 eV was ascribed to sulfide. It is not possible to distinguish between the different sulfides, *i.e.* between CoS_2_ and FeS_2_. Other two contributions centered at 162.9 and 164.5 eV were ascribed to terminal and central sulfur atoms in polysulfides.^39^ There was also a minor contribution from sulfite at 166.7 eV. Compared to the surface spectra, the spectrum after etching did not contain any sulfate contribution.

The XPS region of Co 2p_3/2_ (Fig. 2b and Table 1 column “Co 2p [at%]”) showed a sharp maximum at 778.6 eV with a tail towards the higher binding energies and a minor signal corresponding to the satellite structure at approximately 785 eV. Deconvolution of the Co 2p signal is rather complicated. The original CoS_2_ powder (ESI S1 and S2†) was measured to obtain the best fitting parameters for sulfide. The majority of cobalt sulfide was confirmed by the peak at 778.6 eV. The minor contribution of cobalt oxide suggested a peak at 779.9 eV. The oxide fitting parameters were derived based on detailed literature. During the fitting process, the Co_3_O_4_ signal shape was determined as the most likely oxide compound present in the sample, with a peak at 779.9 eV.^40^ The XPS signal of the Fe–Co–S deposit after etching was fitted by sulfide and oxide fitting patterns, showing a clear majority of sulfidic cobalt and less than 10 rel.% of cobalt oxide. The Fe 2p spectrum (Fig. 2c and Table 1 column “Fe 2p [at%]”) did not show any sign of a sulfide peak pattern. Only the Fe_2_O_3_ pattern was observed on the surface. After the etching, Fe_2_O_3_ pattern with two overlapping Fe 2p_3/2_ peaks at 708.6 and 711.0 eV together with satellite contribution at 730.5 pointed out Fe^2+^ enrichment which is, however, normal for Fe_2_O_3_ samples undergoing etching process.^40^ In summary, XPS analysis provides compelling information that the Fe–Co–S deposit exhibits sulphates contributions only in the superficial layer while after etching, only sulfidic sulfur has been detected. Interestingly, cobalt (or bimetallic Fe–Co) sulfide is a dominant sulfide compound, in contrast to iron, which appears to be completely oxidized.

**Fig. 3:**
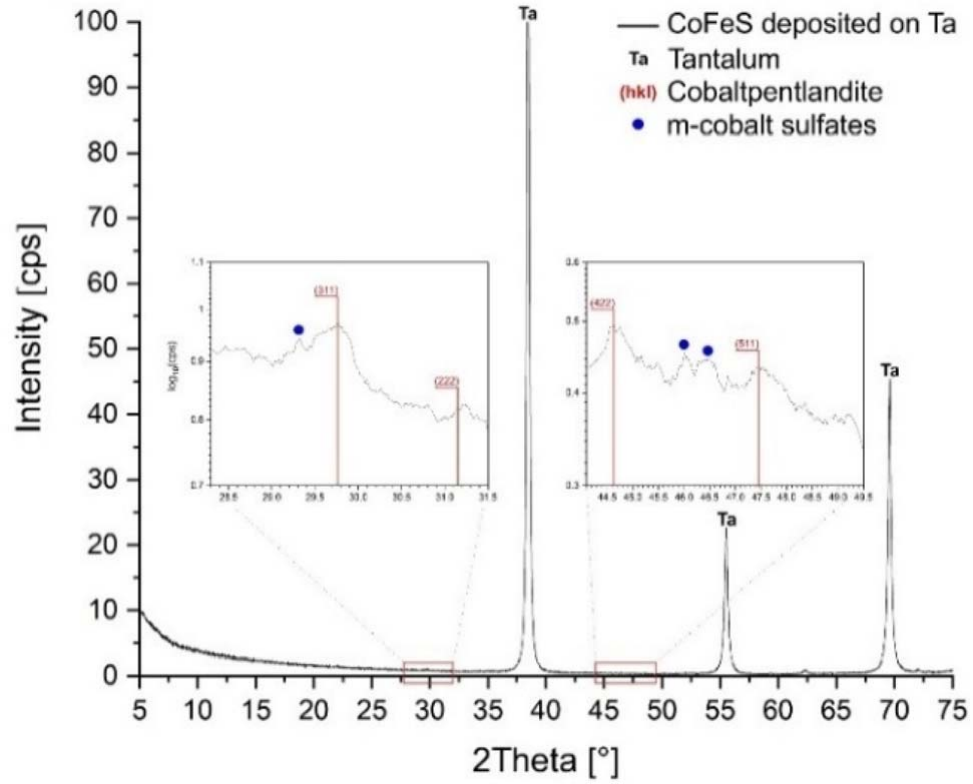
XRD patterns of Fe–Co–S deposit on Ta.

X-ray diffraction of the Fe–Co–S deposit (Fig. 3) predominantly shows diffraction lines of cubic tantalum originating from the substrate. However, upon closer inspection of the XRD spectra, cobaltpentlandite [FeCo_8_S_8_] (PDF 04-005-0440; 2.99–2.86–2.28–1.91–1.75 Å)^63,64^ was also detected (together with minor signals attributed to monoclinic hydrated Co sulfates, marked as m-cobalt sulfates in Fig. 3).

#### 3.2.2 Microscopic characterization of Fe–Co–S deposit

**Fig. 4:**
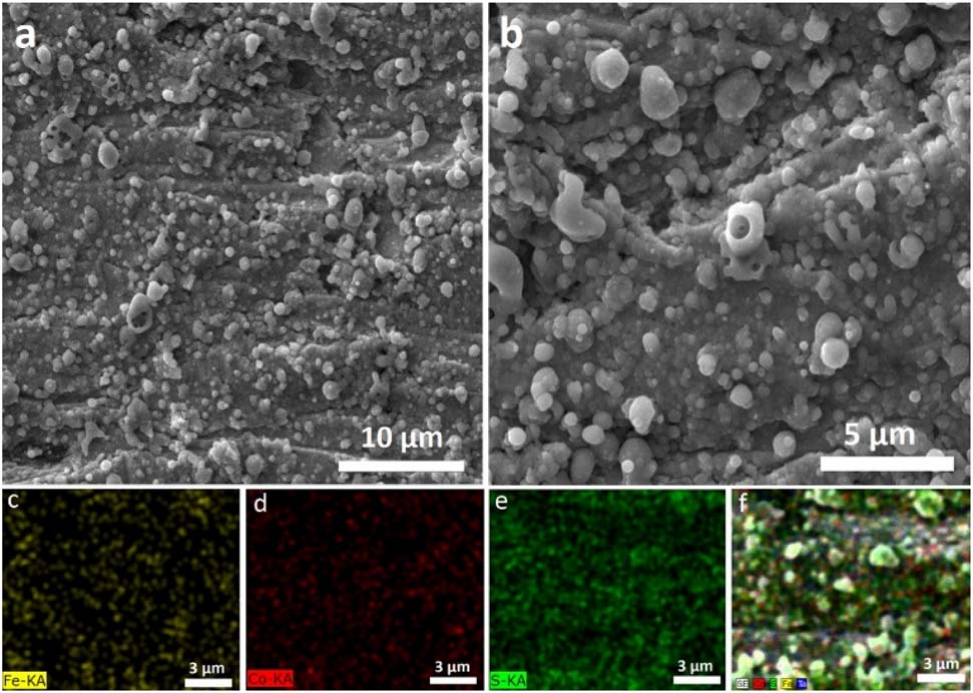
SEM images of the Fe–Co–S deposits on Ta (a) and (b) and its elemental mapping (c)–(f).

SEM images (Fig. 4a and b) show predominantly round particles with sizes of approximately 1 μm and sub-μm. Some of the particles exhibited interesting perforated and/or hollow morphologies. These features are in accordance with vaporized and plasma-produced clusters ejected from the target surface and quenched upon deposition as already observed and investigated in previous related studies.^26,27,29^ Moreover, identical hollow objects have been observed for pulsed laser deposition of hygroscopic compounds,^38^ when laser-induced evaporation and expansion of surface-absorbed moisture of ejected species could result in subsequent solidification of deposited material in the form of hollow morphologies. This assumption is in accordance with here observed hygroscopic behavior of the deposited material (see Section 3.1). The round-shaped objects indicate the rapid cooling of the solidifying gas phase/liquid droplets with unheated Ta surface, which may occur in a metastable state.^26,27,29^ EDS analyses indicated the presence of Fe, Co, S, Ta, and O. The average atomic ratio of the film on Ta was Fe_0.17_Co_0.18_S _0.42_O_0.23_. These values are in line with the partial oxidation of FeS_2_ and/or CoS_2_ and with approximately 1 : 1 ratio of Fe/Co and 1 : 2 of Fe/S and Co/S ratios, which corresponds to the chemical composition of the original irradiated target. Elemental mapping (Fig. 4c–f) depicts the homogenous distribution of tracked elements with sub-micro regions of higher concentration, which indicates the distribution of the particles.

**Fig. 5:**
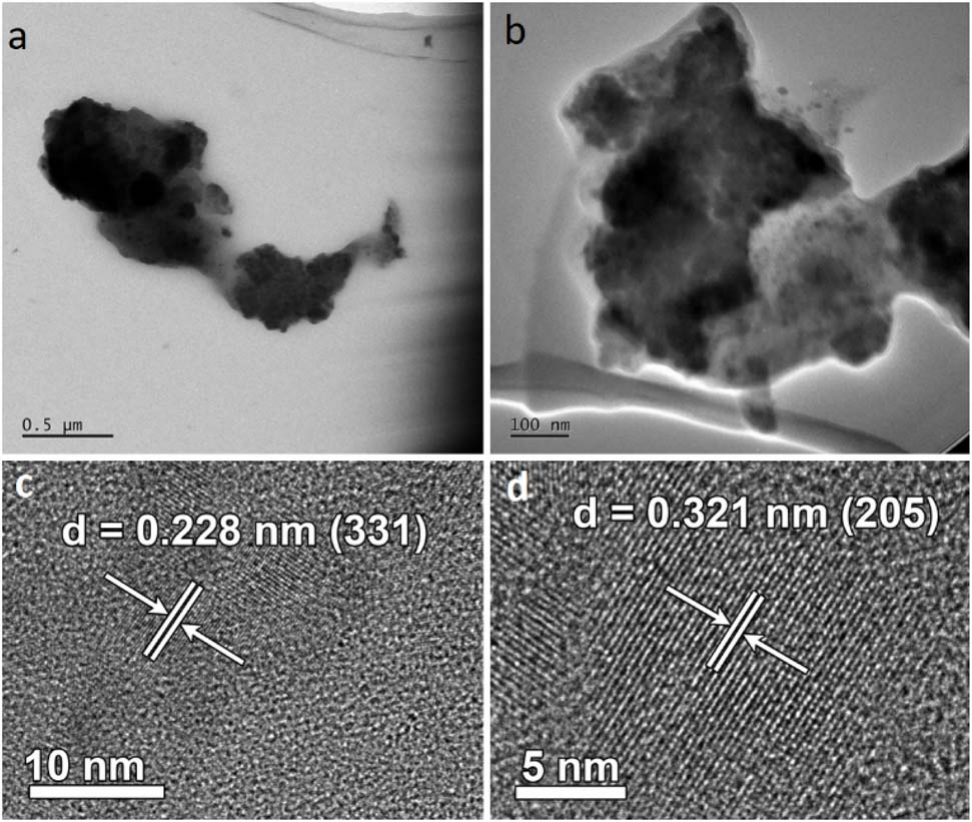
TEM images of Fe–Co–S particles (a) and (b) and HRTEM images depicting interlayer spacing of FeCo_8_S_8_ (c) and γ-Fe_2_O_3_ (d).

TEM images of the Fe–Co–S coat provide more detailed insight into the morphology of the deposit obtained by laser ablation of the FeS_2_–CoS_2_ target. Fig. 5a and b shows irregular agglomerates in the size range of hundreds of micrometers, which consist of smaller spherical nanoparticles sized around tens and units of nanometers, which are embedded in an amorphous matrix. High-resolution electron microscopy image analyses revealed nanocrystalline regions whose interlayer spacing *d* = 0.228 (331) fits with the cubic cobaltpentlandite (PDF 04-005-0440)^63,64^ (Fig. 5c), and the nanoobjects with crystalline spacing *d* = 0.321 nm (205) corresponding to the tetragonal maghemite [γ-Fe_2_O_3_] (PDF 015-0615)^65^ (Fig. 5d). These TEM and HRTEM data were in agreement with the Raman and X-ray spectroscopies.

### 3.3 Plausible mechanism of FeCo_8_S_8_ phase formation induced by pulsed laser ablation

To propose the mechanism of the bimetallic sulfide FeCo_8_S_8_ phase we should consider (i) the original structure of the CoS_2_–FeS_2_ target, (ii) the processes occurring during pulsed laser deposition in a gaseous plume, and (iii) the cooling collision of excited ejected species with an unheated substrate. Raman spectroscopy and XRD revealed that the pristine and irradiated targets consisted of pyrite, cattierite, rhomboclase and cobaltkieserite. During pulsed laser ablation the irradiated phases undergoes chemical, phase and structural transformations, considering that the final Fe–Co–S deposit composed of partially amorphous phase and partially crystalline nano-entities where complementary analyses revealed the presence of cobaltpentlandite, maghemite, minor amount of cobalt oxide and only very superficial contribution of cobalt sulfates. Thermodynamic non-equilibrium high-temperature reactions that could occur during pulsed laser ablation on the irradiated target, which occur *via* interaction of ejected highly energized ionized species in the gas phase are favorable for the deposition of materials with a morphology and phase composition that is different from their bulk progenitors. Thus, the formation of cobaltpentlandite along maghemite, a minor amount of cobalt oxide, and an amorphous phase could be attributed to the gaseous interaction of the ejected excited Fe, Co, and S species, which subsequently collided with the unheated Ta substrate. The formation and interaction of inorganic cluster ions in the plume generated by laser ablation have been thoroughly studied, for example, for metal oxides, phosphides, chalcogenides, and metal carbonyls.^41–45^ Our recent studies described pulsed laser ablation of individual FeS_2_ (ref. 26 and 27) and CoS_2_ (ref. 29) on a Ta substrate (considered as an inert substrate), where the deposition of the original pyrite and cattierite (cubic CoS_2_ phase) took place. Interestingly, in the case of pulsed laser irradiation of the FeS_2_–CoS_2_ mixture, complementary analysis confirmed that the resulting deposit did not contain the FeS_2_ phase but was composed predominantly of cobaltpentlandite along the maghemite. It is questionable whether iron was detected only in the form of its oxide and incorporated into the cobaltpentlandite. Owing to the lack of theoretical models and calculations for such highly thermodynamically non-equilibrium states, we can only state the final products of the reaction and discuss the possible kinetics of a given event. The main factors that could represent the clue for the explanation of given mechanism are proposed to be higher decomposition temperature of CoS_2_ at 650 °C compared to decomposition temperature at 550 °C of FeS_2_,^46,47^ whereas the chemical affinity of Co to S seems to be higher than between Fe and S.^47^ Considering ∼100 °C higher thermal stability of CoS_2_ compared to FeS_2_ we can propose that during collision of excited gaseous Fe, Co, S species with unheated Ta substrate, Co and S in the given ratio could exhibit tendency for deposition of CoS_2_ at higher temperature compared to Fe and S to create FeS_2_ phase. The former desublimation of CoS_2_ in the presence of iron species could result in the incorporation of iron atoms into CoS_2_ and the formation of bimetallic cobaltpentlandite, while residual iron from still decomposed FeS_2_ (due to disruption of the original stoichiometry) did not re-bond to sulfur, but was deposited in an atomic form, which was subsequently oxidized.

**Fig. 6:**
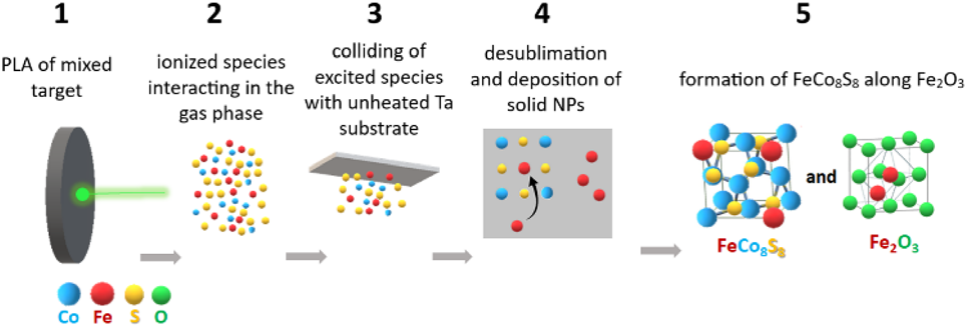
Simplified scheme of the proposed kinetics of laser induced FeCo_8_S_8_ and Fe_2_O_3_ reactive deposition: pulsed laser irradiation of FeS_2_ and CoS_2_ mixture target (1), laser-induced formation of ionized excited Fe, Co, S species interacting in the gas phase (2), colliding of excited Fe, Co, S species with unheated Ta substrate (3), former tendency of CoS_2_ for the deposition in the presence of Fe excited species which results in inclusion of Fe atoms into depositing CoS_2_ (4), formation of FeCo_8_S_8_ and deposition of atomic iron, which oxidized into Fe_2_O_3_ (5).

Simplistic scheme of proposed mechanism is given in Fig. 6.

The final step (4 to 5 in Fig. 6) of above proposed reactive process is very rapid. Optical emission spectroscopy (OES) of plasma induced by ablation of pure iron sulfide performed under related conditions revealed the plasma emission with a lifetime of around 500 ns.^46^ The collision of excited ionic species with unheated Ta surface inevitably results in acceleration of plasma cooling which leads us to assumption that the reaction which took place is concerted. The speed of the process acts in a suppressive manner towards the formation of eventual intermediates, however in the previous step (step 3: intermixing of highly excited species in the plasma state) a transient may be formed. This assumption is supported by previous studies on pulsed laser-induced reaction of gaseous reactants (1,3-disilacyclobutane and dimethyl selenide) where LIF (Laser Induced Fluorescence) experiment allows detection of transient gaseous SiGe, whose radiative lifetimes was assigned 17–20 ns.^66^ Deeper insight into the direct connection between transient phenomena in the plasma state and the final structure formed by the de-sublimation of plasma species onto the substrate will require detailed OES and LIF experiments.

### 3.4 Computational analysis

In order to achieve insight into the thermodynamic stability and an electronic structure of examined phases and thus clarified more deeply mechanism of FeCo_8_S_8_, DFT computational analysis have been performed and described below.

#### 3.4.1 Formation energy

In DFT, formation energy is an important parameter that plays a key role in understanding the relative stability of different atomic substitutions in crystal structures and chemical reactions. The formation energies of Co_0.99_Fe_0.01_S_2_ and Fe_0.99_Co_0.01_S_2_ were calculated according to eqn (1) and (2):1
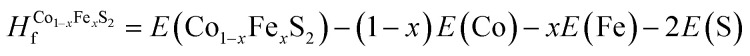
2
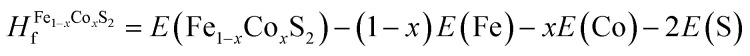
where *E*(Co_1−*x*_Fe_*x*_S_2_) is the total ground state energy of Co_1−*x*_Fe_*x*_S_2_ and *E*(Co), *E*(Fe), and *E*(S) are the total ground state energies of individual Co, Fe, and S atoms, respectively, in their standard configuration. The calculated formation energy of Co_1−*x*_Fe_*x*_S_2_ per atom was −0.10138 eV where in Fe_0.99_Co_0.01_S_2_ the calculated formation energy was −0.01364 eV.

#### 3.4.2 Magnetic properties and electronic structure

**Fig. 7:**
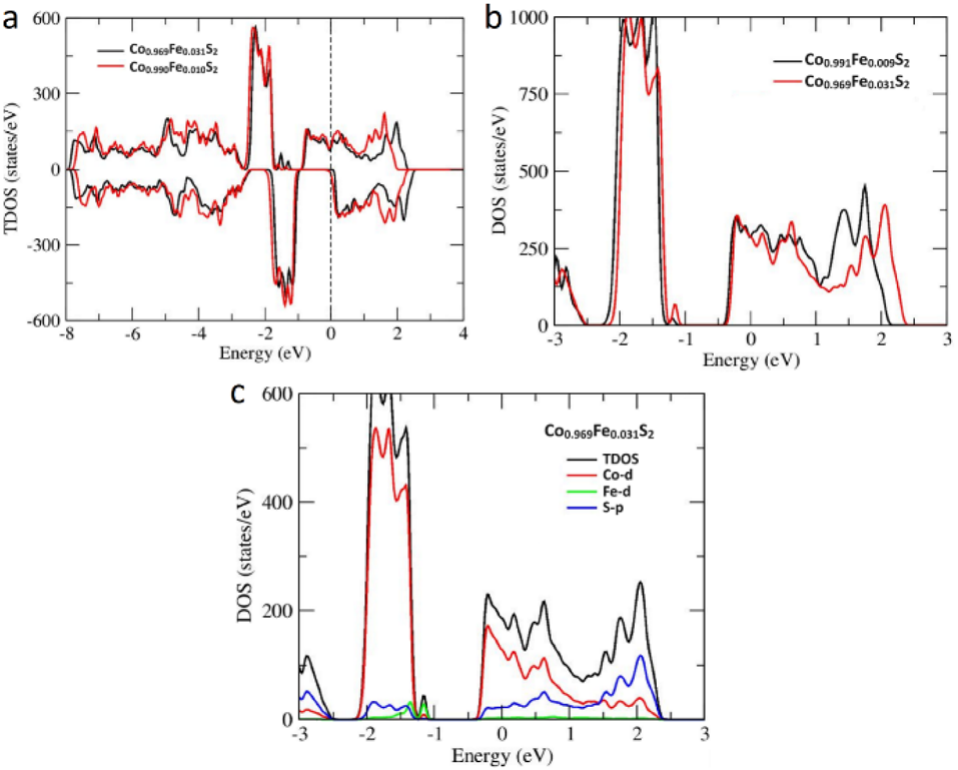
Effect of Fe doping on DOS of Co_1−*x*_Fe_*x*_S_2_ with different concentration (a), calculated total density of states of Co_1−*x*_Fe_*x*_S_2_ (*x* = 0.009, 0.031) in the paramagnetic state (b), angular-momentum-projected density of states of Co_1−*x*_Fe_*x*_S_2_ (*x* = 0.031) in the paramagnetic state (c).

To explore the magnetic nature of CoS_2_, Co_1−*x*_Fe_*x*_S_2_ and FeS_2_, in DFT, the total energies from the ground state calculations were compared for different magnetic states, as shown in Table 2. The magnetic state can be represented according to the orientation of the spin configurations in the ferromagnetic, antiferromagnetic or paramagnetic states. The configuration with the lowest total energy corresponds to the ground state of the FeS_2_‚ CoS_2_, Fe_1−*x*_Co_*x*_S_2_ and Co_1−*x*_Fe_*x*_S_2_ that provide insights into its magnetic properties. The energy difference between FeS_2_ and Fe_1−*x*_Co_*x*_S_2_ is approximately 0.2 meV likely to exhibit a paramagnetic state, while CoS_2_ and Co_1−*x*_Fe_*x*_S_2_ are ferromagnetic at 0 K with a half-metallic nature. It is characteristic of Co_1−*x*_Fe_*x*_S_2_ that density of states (DOS) has a dominant contribution at the Fermi energy constructed only from the spin-up band, while the spin-down state shows a gap revealing a half-metallic spin state (see Fig. 7a). The localized kinks in the gap of the spin-up DOS formed by the Fe 3d state are slightly shifted to higher energies from Co_0.99_Fe_0.01_S_2_ to Co_0.969_Fe_0.031_S_2_ but the effect on the band gap in spin-down channel is negligible. The magnetic moment is produced by the Co-3d and Fe-3d states as Co = 0.889 *μ*_B_, Fe = 0.266 *μ*_B_ and *S* = 0.031 *μ*_B_. The magnetic moment at the S site was mainly induced by the Co-3d electron. According to the literature the Curie temperature of CoS_2_ is 122 K.^48^

**Table 2:** Ground state energy of paramagnetic state minus ground state energies of different magnetic configuration

Spin configuration	Ground states energies in (eV)			
	FeS_2_	CoS_2_	Fe_0.99_Co_0.01_S_2_	Co_0.99_Fe_0.01_S_2_
Paramagnetic	0.0000	0.0000	0.00000	0.0000
Ferromagnetic	0.0002	0.8340	−0.00001	−4.0904
Antiferromagnetic	0.0005	0.2683	−0.00013	−0.9861

Based on Curie temperature of CoS_2_ the electronic structure calculation of Co_1−*x*_Fe_*x*_S_2_ were performed in paramagnetic phase. Fig. 7b shows the paramagnetic total DOS of Co_1−*x*_Fe_*x*_S_2_ (*x* = 0.009 and 0.031). Increasing the Fe concentration in CoS_2_ slightly shifts the bandwidth (−2.2 to −1.0 eV), whereas the shift below the Fermi energy (*E*_F_) is hardly visible. This is because the contribution of the Fe 3d state is very small around the Fermi energy, as shown by the angular-momentum-projected density of states (PDOS) of Co_1−*x*_Fe_*x*_S_2_ (*x* = 0.031). The DOS around *E*_F_ is mainly constructed from Co-3d states and S-3p states and Fe-3d states do not influence the Co-3d and S-3p state (see Fig. 7c). The total DOS Fe_0.99_Co_0.01_S_2_ show semiconducting nature having the band gap of 1.3 eV (see Fig. S5 in ESI†).

#### 3.4.3 Discussion of computational analysis

The aim of DFT part was to obtain reliable quantitative information regarding the structural stability and electronic structure. Based on the calculated formation energies a deeper understanding of the thermodynamic stability of Fe in Co_1−*x*_Fe_*x*_S_2_ is possible, while Co in Fe_1−*x*_Co_*x*_S_2_ is less stable. Therefore, our theoretical prediction provides evidence for why the pulsed laser irradiation of the FeS_2_–CoS_2_ mixture does not contain the FeS_2_ phase but is mainly composed of cobaltpentlandite along with the maghemite (see Section 3.2). The Fe bonded with O is thermodynamically more stable than that bonded with S. Additionally, the electronegativity difference of Fe–O is greater than that of Fe–S, which also favors Fe–O ionic bonding in FeS_2_–CoS_2_ mixture. The increasing Fe concentration in Co_1−*x*_Fe_*x*_S_2_ was carefully checked (see Fig. S6 in ESI†) which intensifies the Fe-3d from localized to delocalized and merged with a bandwidth from −1.2 to −2.2 eV but the DOS around *E*_F_ is unchanged which provide a clue that at equilibrium Fe concentration (up to 10%) does not change the electronic structure significantly and Co_1−*x*_S_2_ stay half-metallic. The photocatalytic measurement of Co_1−*x*_Fe_*x*_S_2_ is close to that of CoS_2_ (half-metallic) compared to FeS_2_ (semiconductor), which shows that the present DFT calculations are in agreement with the experimental results. To demonstrate the application potential of the achieved Fe–Co–S deposit, photo-, Fenton-like and electrochemical catalytic tests were performed and are described in the following sections.

### 3.5 Catalytic and electrochemical performance of Fe–Co–S deposit

The catalytic activity of the Fe–Co–S deposit on the Ta substrate was examined by MB degradation in the terms of Fenton-like process and the photocatalytic process (under daylight exposure) in comparison with the single FeS_2_ and CoS_2_ deposits. MB dye was selected for primary catalytic tests as the model standard dye in aqueous solution to demonstrate the primary degradation ability of the organic compounds.

#### 3.5.1 Fenton-like catalytic activity for MB degradation

**Fig. 8:**
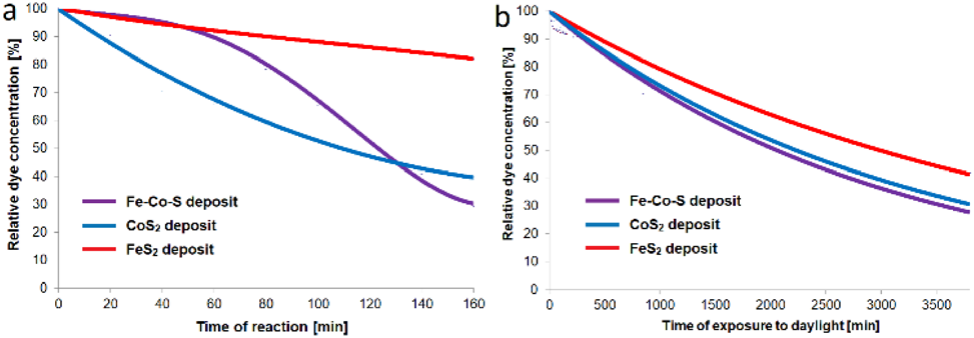
Comparison of MB depletion under the conditions of Fenton like degradation (a) and day light driven degradation (b) in the presence of Fe–Co–S, CoS_2_, FeS_2_ deposits.

After immersion of the examined deposits into MB solutions, small bubbles were generated on the surfaces, suggesting an ongoing Fenton-like degradation process. The temporal evolution of the MB degradation measured by UV-Vis spectrometer (see the conditions in experimental) for Fe–Co–S compared to individual FeS_2_ (ref. 26 and 27) and CoS_2_ (ref. 29) deposits for the Fenton-like process and solar light driven decomposition is depicted in Fig. 8a and b. The MB decomposition in terms of the Fenton-like process was monitored for 3 hours (180 minutes), whereas the spectra starts to be recorded after 20 minutes in order to filter out the effect of MB adsorption onto the Fe–Co–S coat (Fig. 8a). The degradation of MB after 180 minutes corresponds to residual relative concentration of 82% for FeS_2_ deposit, 40% for CoS_2_ deposit and 30% for Fe–Co–S deposit. The depletion of MB reflects MB degradation owing to the absence of an adsorption peak at 256 nm, assignable to the leuco-MB form. UV-Vis spectra of MB depletion are available in the supplemental data (Fig. S7† – for FeS_2_; Fig. S8† – for Fe–Co–S; in supplemental data in ref. 29 for CoS_2_). The activities of the FeS_2_ and CoS_2_ phases for Fenton-like degradation processes have been described in previous studies.^29,49^ To the best of our knowledge, the Fenton-like activity of bimetallic FeCo_8_S_8_ has not been examined thus far, even though Fe ions,^50^ zero-valent Fe^51^ and cobalt ions^52^ have been broadly studied for such processes. Fe–Co–S based coat exhibits significantly higher efficiency for Fenton-like degradation of MB compared to the FeS_2_ and CoS_2_ coat. The mechanism of synergistic effect between different metals in bimetallic systems and their complexes which leads to enhanced heterogeneous Fenton-like performance has been broadly studied for bimetallic oxides^53^ and so far attracted less attention for bimetallic sulfides.^54^ The enhancement of degradation activity of synergistically acting bimetallic system is attributable especially to: (i) significant increasing of the active sites on the surface, (ii) accelerating the transfer of electrons and the redox cycle of metal ions which occurs due to the interaction between the oxidation–reduction pairs of iron and cobalt. This redox process can temporarily slow the radical formation and MB decomposition in the initial stage (observed in the first 120 minutes – Fig. 8a violet curve). Consequently, partial surface oxidation of sulfides to sulfates occurs simultaneously with the catalysis of primary radicals, which results in gradual production of sulfate ions and in acceleration of the reaction in later stage.^67^ Thus well-designed bimetallic oxide and/or sulfide system and their complexes are favourable for forming ˙OH from H_2_O_2_ in a wide pH range and provide higher Fenton-like efficiency for organics degradation and also using of H_2_O_2_ with limited metal ion leaching and prolonged stability. However, in our case also the contribution of presence of the Fenton-active Fe_2_O_3_ (ref. 55) and/or amorphous phase and its potentially synergistic interactions with dominant FeCo_8_S_8_ phase may have an additional effect.

#### 3.5.2 Photocatalytic MB degradation under day light

The photocatalytic activity of the same 3 coats have been tested under natural day light. The conditions of the photo-catalytic test corresponded to normal, less intensive sunlight (average intensity of sunlight: *Φ* ∼ 700 lx). Despite such adverse conditions for the photocatalytic process (low sunlight intensity and low surface area of the active area compared to the MB solution volume), the degradation of MB progressed (Fig. 8b). After approximately 2.5 days (3700 minutes), the relative concentration of MB decreased to 82% for FeS_2_, 40% for CoS_2_ and 30% for Fe–Co–S deposit (UV-Vis spectra of photocatalytic MB decrease are available in supplemental data Fig. S9–S11†). The photocatalytic performances of CoS_2_ (ref. 56) and FeS_2_ (ref. 28) have been broadly studied. Bimetallic cobalt–iron sulfide is considered to possess superior photocatalytic efficiency compared to monometallic CoS_2_,^19^ which agrees with the results obtained under very mild conditions. In accordance with previous studies on bimetallic sulfide photo-catalysts the key specificities of FeCo_8_S_8_ presented in Fe–Co–S deposits that lead to an increase in photocatalytic activity are assignable to: faster photo-generated electron transfer and also more convenient trajectories of electron and hole transfer^57^ accompanied by the bimetallic systems tendency to exhibits reducing of band gap value and enhancing visible light response (compared to its monometallic counterparts).^57^ Additionally, the synergistic effect between nanostructured FeCo_8_S_8_ and Fe_2_O_3_ should be taken into account, as strongly enhanced photocatalytic performance has been described for the related system of FeS_2_/Fe_2_O_3_ composite.^58^

#### 3.5.3 Solar-light driven photocatalytic activity for degradation of selected antibiotics

**Fig. 9:**
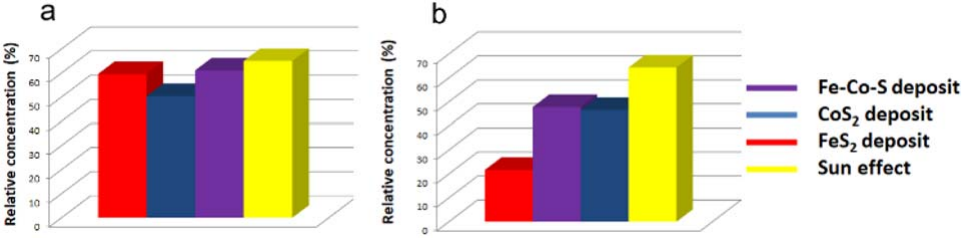
Solar light driven degradation of sulfamethoxazole (a) and trimethoprim (b) showing relative residual concentration after 29 hours of sunlight irradiation in absence of deposits (pure sunlight effect) and in the presence of Fe–Co–S, CoS_2_, FeS_2_ deposits on Ta.

The Fe–Co–S deposit on Ta, which demonstrated superior photocatalytic efficiency for MB degradation compared to FeS_2_ and CoS_2_, has been evaluated for its solar light-driven photocatalytic efficiency in the degradation of sulfamethoxazole (SMX) and trimethoprim (TMP). These compounds were selected as representative antibiotics whose residues are frequently detected in wastewater.^59–61^ The conditions remained the same as for the MB photocatalytic degradation described above (see Section 2.3) but the intensity of the simulated solar light used here was higher (100 klx). Fig. 9 show progress of SMX and TMP depletion respectively. Both antibiotics slowly degrade also under the influence of solar light irradiation when slightly higher degradation degree after 29 h of sun light exposure has been measured for SMX (relative concentration: 65%) than for TMP (relative concentration: 64%). For both antibiotics the presence of the Fe–Co–S deposit enhanced the degree of degradation. After 29 h of solar light exposure, SMX exhibited relative concentration of 61% and TMP of 47%. A detailed comparative study of SMX and TMP degradation under various AOPs, together with their toxicity and degradation by-products, has been described in the literature.^59–61^ Based on DFT calculations and previous experimental studies, SMX exhibits a higher photodegradation rate than TMP.^59–61^ Therefore, it is notable that in the presence of the Fe–Co–S deposit, TMP undergoes significantly higher degradation compared to SMX. To compare photocatalytic efficiency of mixed Fe–Co–S with its individual counterparts the same test was performed also with CoS_2_ and FeS_2_. A comparison of the influence of different deposits can be clearly seen in Fig. 9 (time-dependent degradation curves are provided in Fig. S12 and S13†). Efficiency of Fe–Co–S for degradation of SMX is comparable with FeS_2_ (61% and 59% of respectively), while the CoS_2_ deposit showed the highest degradation level (50% of residual concentration). In case of TMP effect of Fe–Co–S is almost the same as for CoS_2_ coat (∼47%), whereas significantly higher photocatalytic contributions shows FeS_2_ (22% of residual TMP concentration).

In contrary to Fenton-like and photocatalytic degradation of MB the Fe–Co–S deposit does not exhibit enhanced effect for SMX and TMP.

A detailed explanation of the differential photodegradation effect towards specific antibiotics will require a separate study. It will be necessary to investigate the contribution of individual phases and surface properties (*e.g.* surface charge) of the deposits in relation not only to the given antibiotics, but also to the degradation products.

#### 3.5.4 Electrochemical properties

**Fig. 10:**
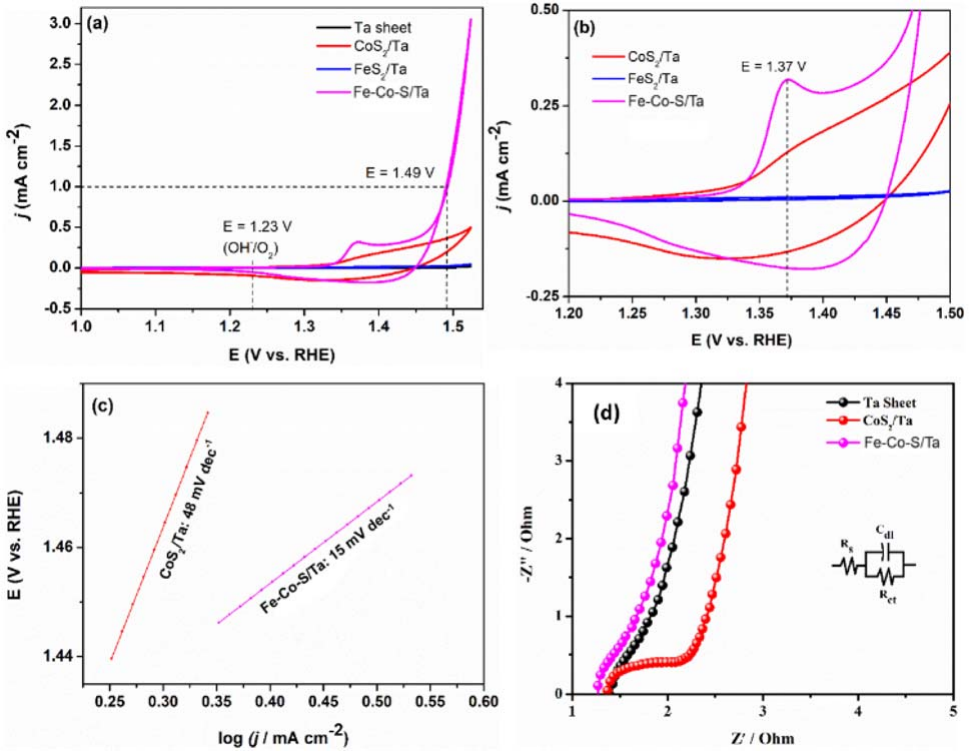
(a) CV curves with a scan rate of 10 mVs^−1^ in 1.0 M KOH solution for the pristine Ta sheet, CoS_2_/Ta, FeS_2_/Ta, Fe–Co–S/Ta, respectively. (b) Magnified view of CoS_2_/Ta, FeS_2_/Ta, Fe–Co–S/Ta voltammetric traces presented in (a). (c) Tafel plots for CoS_2_/Ta and Fe–Co–S/Ta. (d) Nyquist plots of Ta sheet, CoS_2_/Ta and Fe–Co–S/Ta samples measured in the frequency range from 100 kHz to 0.1 Hz with an amplitude of 5 mV (peak-to-peak) at open circuit potential. (Inset: Equivalent circuit used for fitting electrochemical impedance spectra; the fitted EIS plot and the raw data points used for fitting are presented together in Fig. S14.†)

The OER catalytic performance of FeS_2_,^26,27^ CoS_2_ (ref. 29) and Fe–Co–S coats on Ta were evaluated in a typical three-electrode system with an Ar-saturated 1.0 M KOH solution as the electrolyte. The results of the electrochemical studies on all prepared metal sulfides films formed on Ta sheets are presented in Fig. 10. The peak appearing at 1.37 *versus* reference hydrogen electrode (RHE) in Fig. 10a and b refer to the conversion of Co^3+^ to Co^4+^ attributed to the oxidation of CoOOH to CoO_2_.^62^ When scanning to potentials higher than 1.5 V *versus* RHE, the current density rapidly increases due to the OER. The overpotential (*η*) at a current density of 1 mA cm^−2^ was employed to evaluate the electrocatalytic activity for the OER. It can be seen that Fe–Co–S deposited interface show a small overpotential of 260 mV (Fig. 10a) as compared to the individual CoS_2_ deposit. It should be noted that the FeS_2_ deposit and pure Ta sheets exhibited little or no electrocatalytic activity toward the OER within the potential range presented in Fig. 10a. The Fe–Co–S deposited interface exhibits the lowest Tafel slope of 48 mV dec^−1^ in the selected potential range (Fig. 10c) and hence required lower energy to activate water as compared to monometallic CoS_2_ interfaces. Further, the charge transfer resistance of Fe–Co–S deposits was relatively smaller than CoS_2_ (0.8 ohm – Fig. 10d), indicative of the higher electronic conductivity. Taking into fact that FeS_2_ exhibit no significant water oxidation activity, it is plausible to state that the higher-valence cobalt species in the Fe–Co–S deposit coupled with higher electronic conductivity activates water at potential above 1.45 V leading to the faster kinetics towards water oxidation and subsequent evolution of oxygen.

## 4. Conclusions

Despite the many attractive properties of bimetallic iron–cobalt sulfides, which exceed the characteristics of monometallic iron and cobalt sulfides and thus expand the application potential, a facile one-step process for the formation of this desirable phase remains challenging. In this contribution, we report our finding that pulsed laser irradiation of equimolar mixed FeS_2_ and CoS_2_ targets results in the deposition of nanostructured thin films on Ta substrates. The prepared nanostructured deposits were analyzed by scanning electron microscopy, Raman spectroscopy, X-ray photoelectron spectroscopy, X-ray diffraction, high resolution electron microscopy, and electron diffraction. These complementary analyses revealed that the deposit on Ta consisted of nanocrystalline cobaltpentlandite [FeCo_8_S_8_] along the maghemite [γ-Fe_2_O_3_] covered with only a superficial layer of cobalt sulfate. The mechanism of FeCo_8_S_8_ formation is attributed to the intermixing events of gaseous excited Fe, Co, and S species in laser-induced plasma, followed by the higher deposition tendency of CoS_2_ in the presence of iron ions/atoms, leading to the creation of the FeCo_8_S_8_ phase.

The FeCo_8_S_8_-based deposit was tested for its ability to degrade methylene blue under the conditions of daylight photocatalysis and Fenton-like reactions, and for its ability to degrade sulfamethoxazole and trimethoprim under solar light-driven photocatalysis. Catalytic testing showed the superior performance of FeCo_8_S_8_-based deposits for MB degradation compared to individual FeS_2_ and CoS_2_, whereas the Fenton-like activity of bimetallic FeCo_8_S_8_ is revealed here for the first time. Solar light-driven activity of FeCo_8_S_8_-based deposit for degradation of SMX and TMP was not increased compared to individual monosulfides. Detailed explanation requires further separate catalytic study. In addition, the electrochemical properties of the FeCo_8_S_8_-based deposit toward oxygen evolution reaction (OER) were studied and FeCo_8_S_8_ exhibited a significantly higher electrocatalytic alkaline water oxidation activity than FeS_2_ and CoS_2_. DFT calculations involving the investigations on thermodynamic stability and electronic structure of mixed FeS_2_ and CoS_2_ supported the exclusive formation of cobaltpentlandite [FeCo_8_S_8_] along with the maghemite [γ-Fe_2_O_3_] phase upon pulse laser irradiation.

## Author contributions

Tomáš Křenek: conceptualization, data curation, investigation, methodology, visualization, writing – original draft, supervision. Lukáš Vála: conceptualization, writing – original draft, visualization, investigation, data curation, supervision. Palaniappan Subramanian: conceptualization, writing – original draft, visualization, investigation, data curation. Saleem Ayaz Khan: conceptualization, writing – original draft, visualization, investigation, data curation. Ján Minár: data curation, investigation, methodology, supervision. Martin Koštejn: data curation, investigation, formal analysis. Rostislav Medlín: data curation, investigation, formal analysis. Petr Mikysek: data curation, investigation, formal analysis. Věra Jandová: data curation, investigation, formal analysis. Veronika Vavruňková: data curation, investigation, formal analysis.

## Conflicts of interest

There are no conflicts to declare.

## Supplementary Material

RA-015-D5RA03059E-s001

## Data Availability

The source data are available in Zenodo repository with the identifier: https://doi.org/10.5281/zenodo.13853215. Preprint copy available at: http://dx.doi.org/10.2139/ssrn.5169823.
