# Supplementary material for: One step laser-induced synthesis of a bimetallic iron–cobalt sulfide for efficient solar light driven, Fenton-like and electrochemical catalysis
Source: RSC Adv. 2025 Jul 23;15(32):26371–82. doi: 10.1039/d5ra03059e (PMC12284886; doi:10.1039/d5ra03059e)
Supplement: RA-015-D5RA03059E-s001 [file RA-015-D5RA03059E-s001.pdf]

Supplementary Information (SI) for RSC Advances.
This journal is © The Royal Society of Chemistry 2025

SUPPORTING INFORMATION

One step laser-induced synthesis of bimetallic iron-cobalt sulfide for efficient solar light driven, Fenton-like and electrochemical catalysis

Tomáš Křenek [a, b] *, Lukáš Vála [a, b] *, Palaniappan Subramanian [b], Saleem Ayaz Khan [b], Ján Minár [b], Martin Koštejn [c], Rostislav Medlín [b], Petr Mikysek [d], Věra Jandová [c], Veronika Vavruňková [b]

[a] University of West Bohemia, Faculty of Mechanical Engineering, Department of Material and Engineering Metallurgy, Univerzitní 8, 301 00 Pilsen, Czech Republic
[b] University of West Bohemia, New Technologies-Research Center, Univerzitní 8, 306 14 Pilsen, Czech Republic
[c] Institute of Chemical Process Fundamentals of the Czech Academy of Sciences, Rozvojová 135, 165 02
Prague 6, Czech Republic
[d] Institute of Geology of the Czech Academy of Sciences, Rozvojová 269, 165 00 Praha 6, Czech Republic

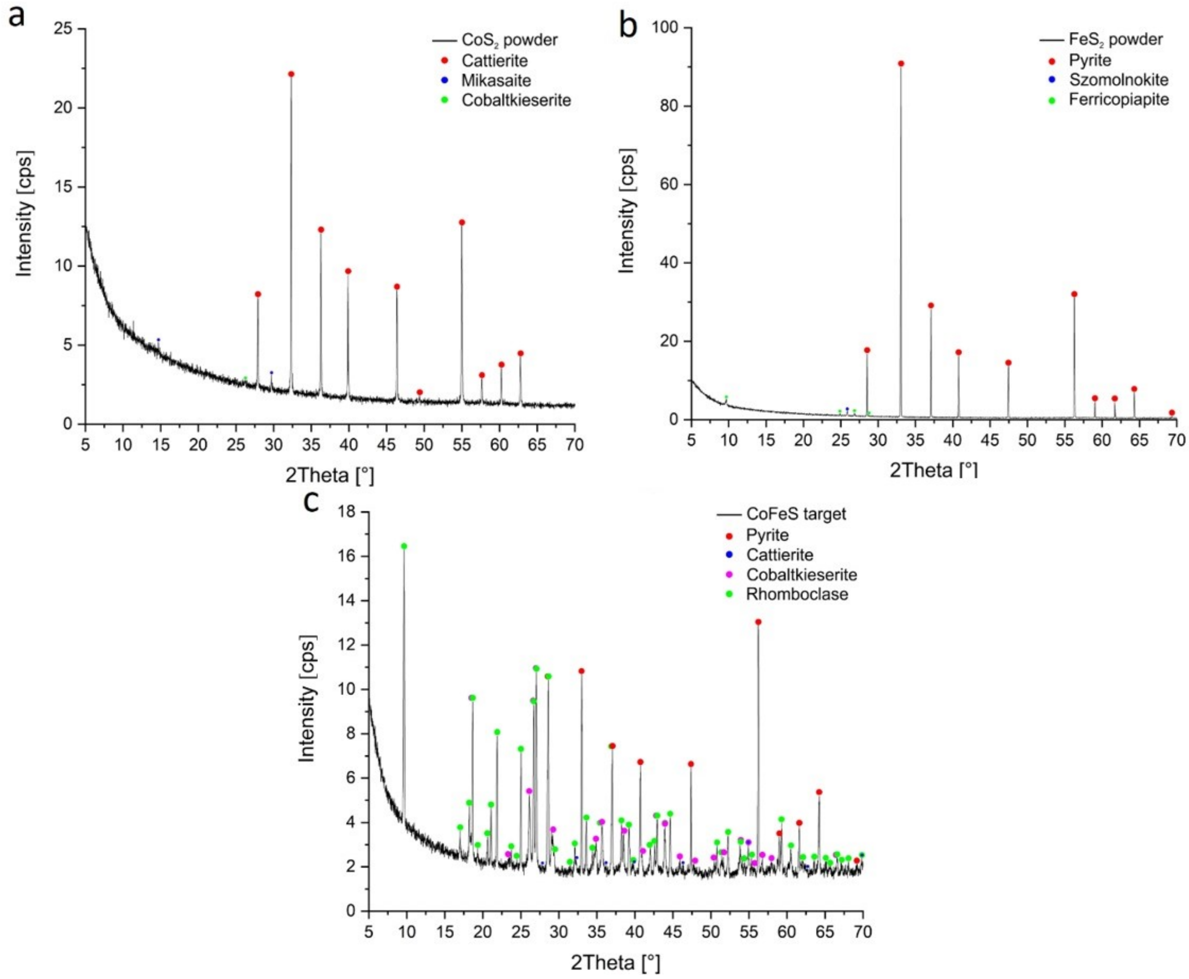

Fig. S1 XRD patterns of original CoS_2 powder (a); original FeS_2 powder (b); pressed target of equimolar CoS_2-FeS_2 powder mixture (c); The depiction of trace phases such as mikasaite (14.73° and 29.74°), cobaltkieserite (26.23° and 35.81°), szomolnokite (25.83° and 28.72°) and ferricopiapite (9.76–24.85–26.83–28.78°) corresponds to the 2Theta angles shown in parentheses. The same is applicable for the cattierite (27.89–32.30–36.24–39.83–46.32°) shown in Fig. 1c

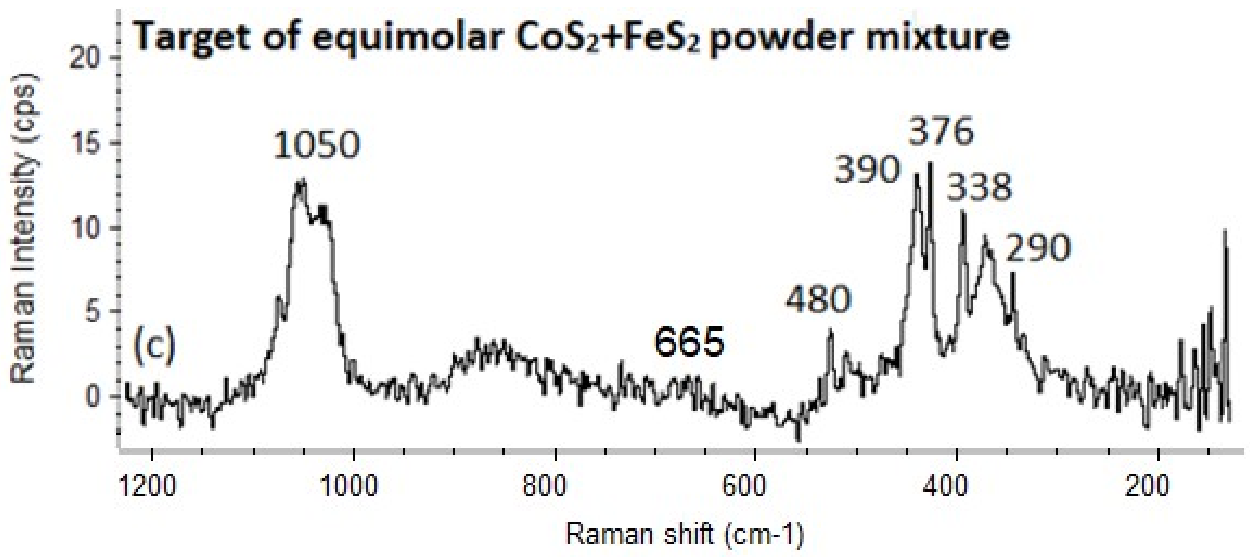

Fig. S2 Raman spectroscopy of target prepared with FeS_2-CoS_2 equimolar powder mixture

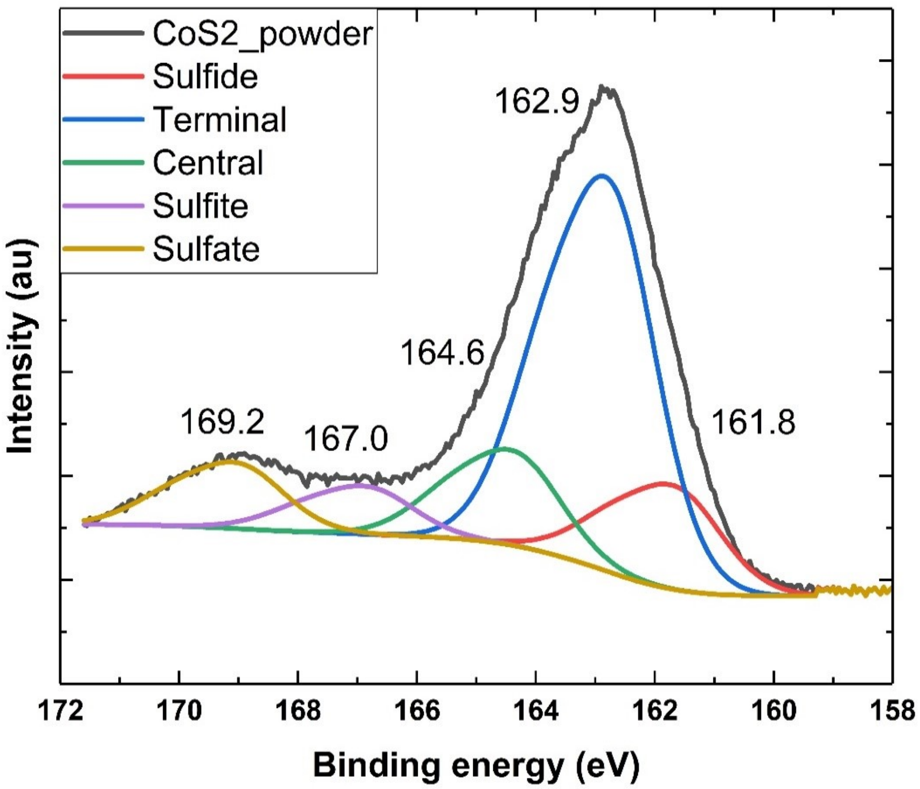

Fig. S3. S 2p spectrum of original CoS_2 powder

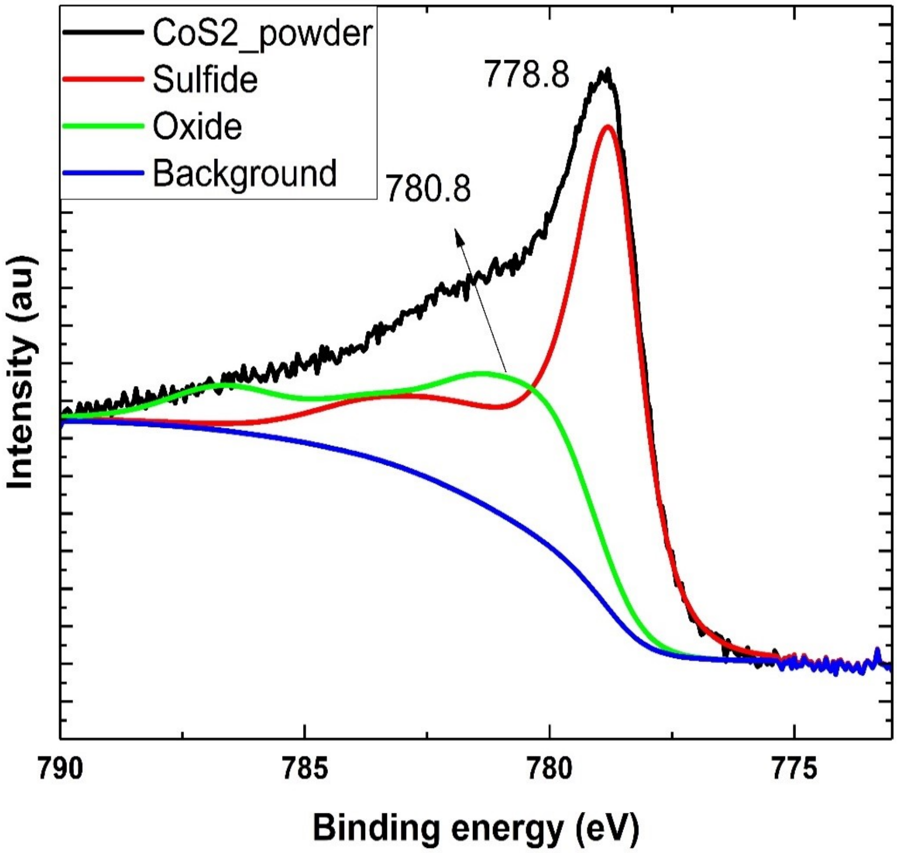

Fig. S4. Co 2p 3/2 spectrum of original CoS_2 powder

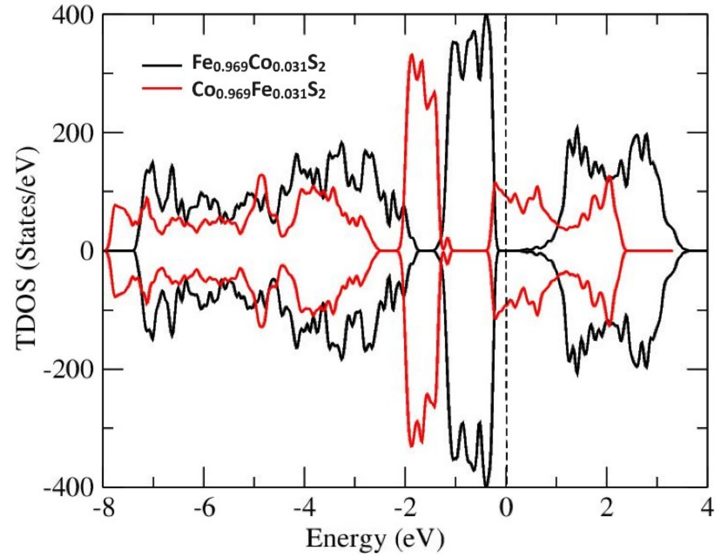

Fig. S5. DOS of $Co_{1-x}Fe_xS_2$ and $Fe_{1-x}Co_xS_2$ with (x= 0.031)

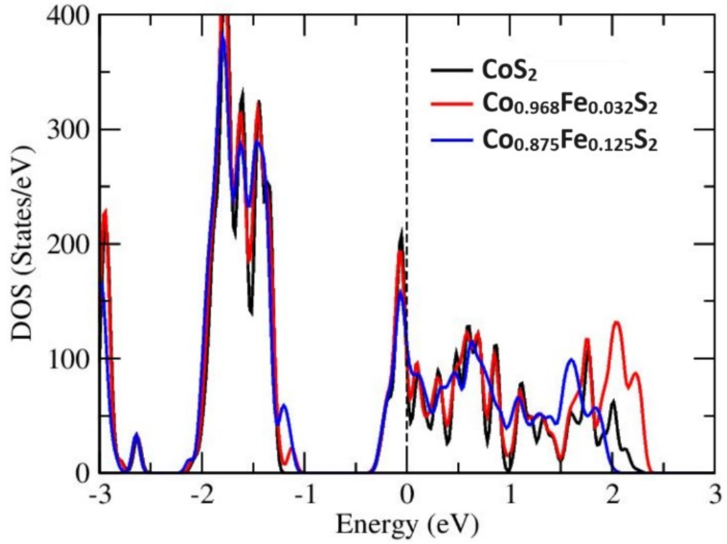

Fig. S6. DOS of $Co_{1-x}Fe_xS_2$ (x= 0.0, 0.033, 0.125)

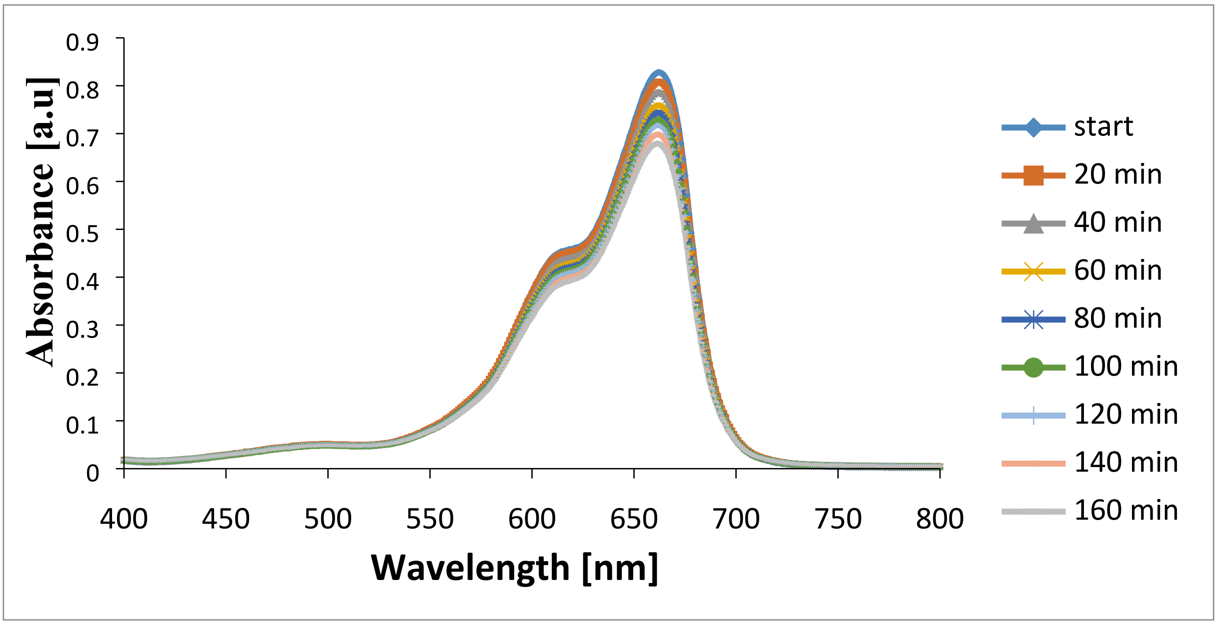

Fig. S7. Progress of Fenton-like driven degradation of MB in presence of FeS_2 deposit

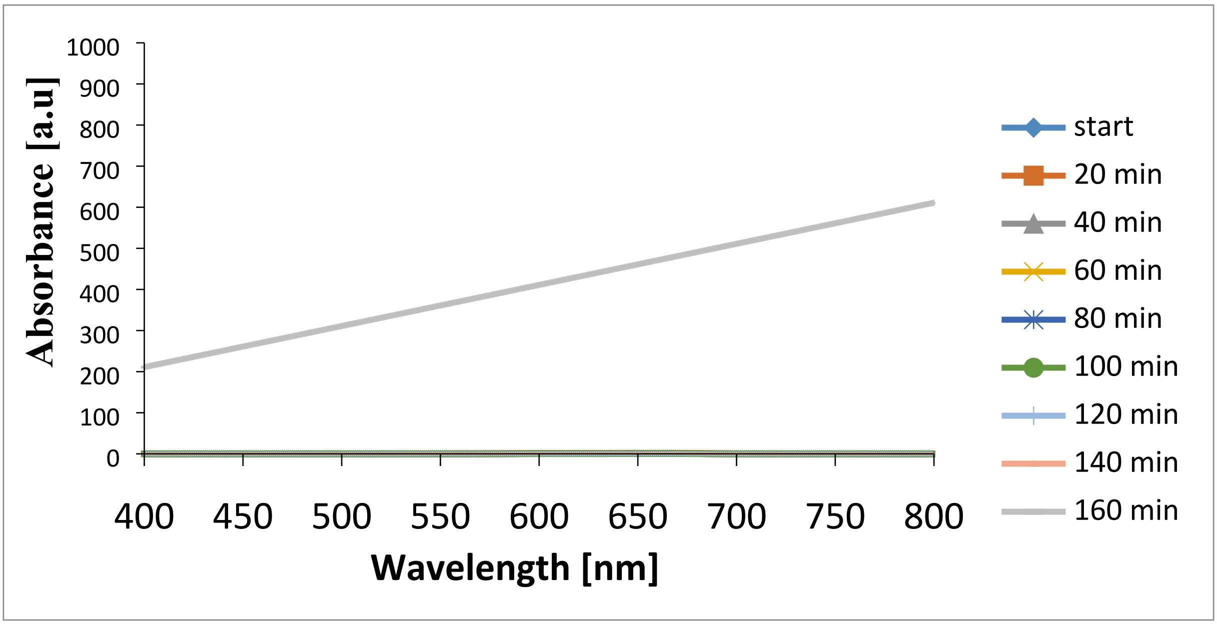

Fig. S8. Progress of Fenton-like driven degradation of MB in presence of Fe-Co-S deposit

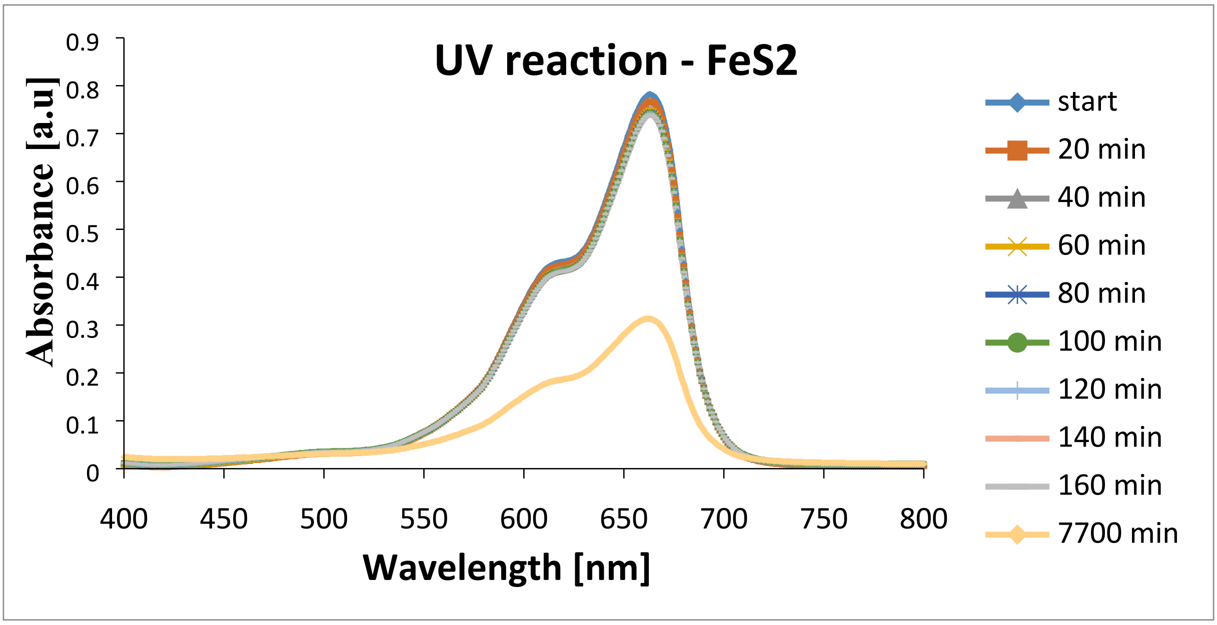

Fig. S9. Progress of daylight driven degradation of MB in presence of FeS_2 deposit

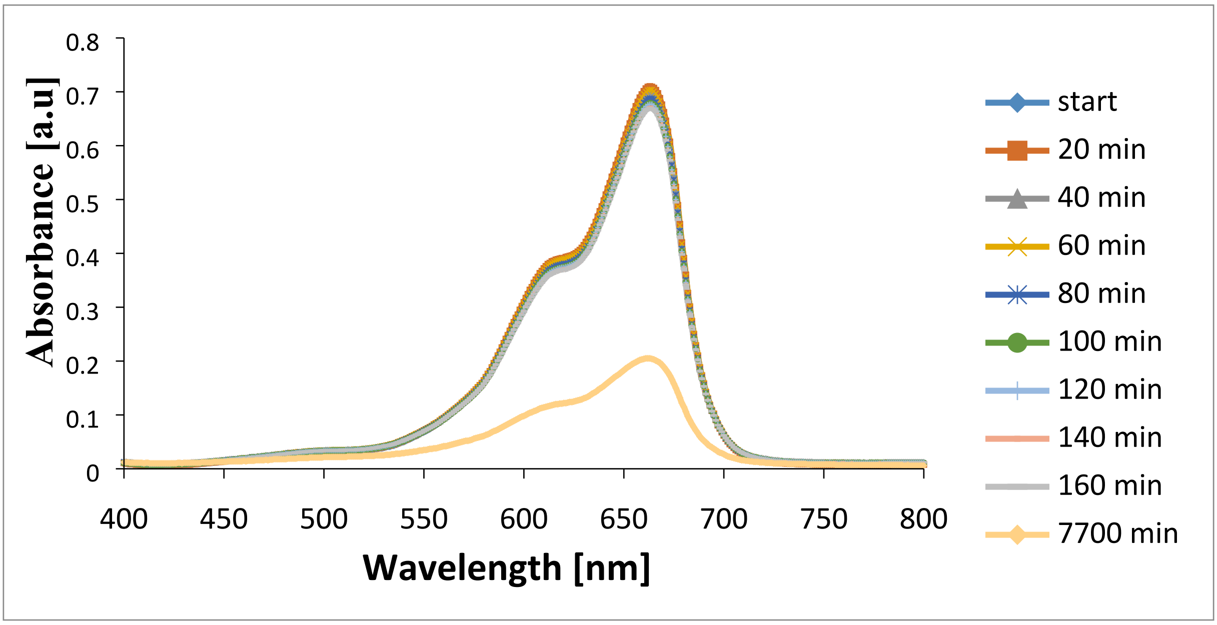

Fig. S10. Progress of daylight driven degradation of MB in presence of CoS_2 deposit

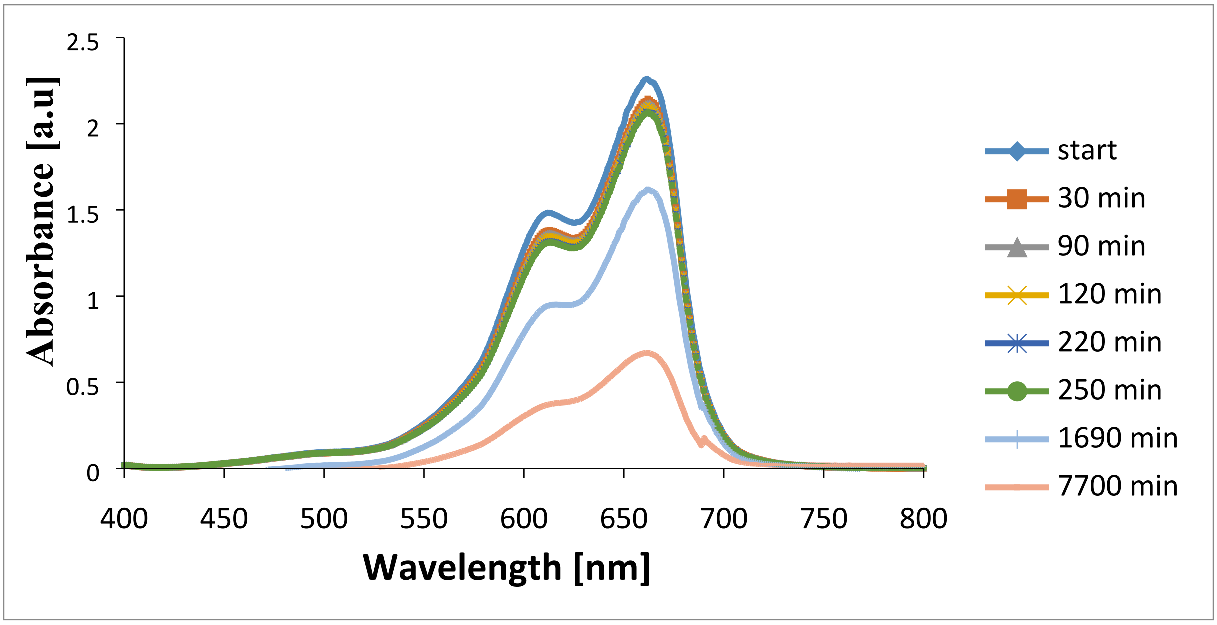

Fig. S11. Progress of daylight driven degradation of MB in presence of Fe-Co-S deposit

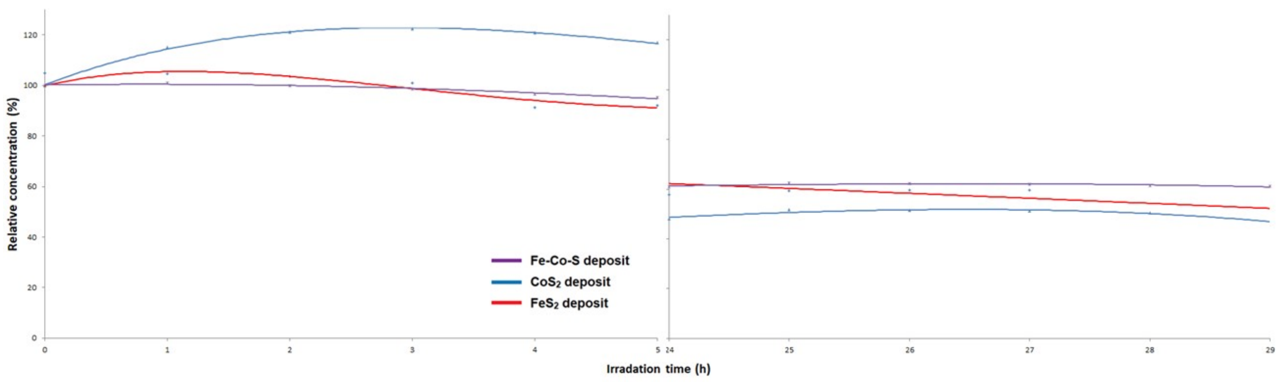

Fig. S12. Progress of solar-light driven photocatalytic activity for degradation of SMX

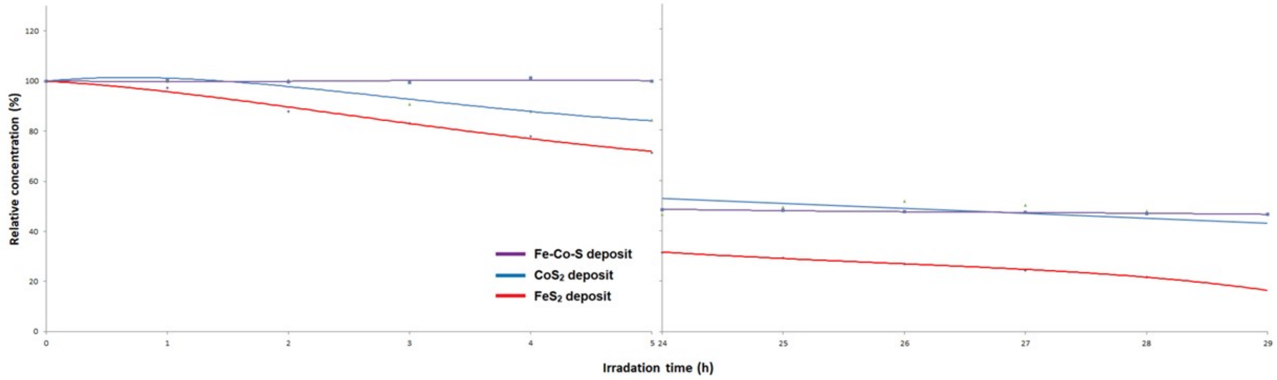

Fig. S13. Progress of solar-light driven photocatalytic activity for degradation of TMP

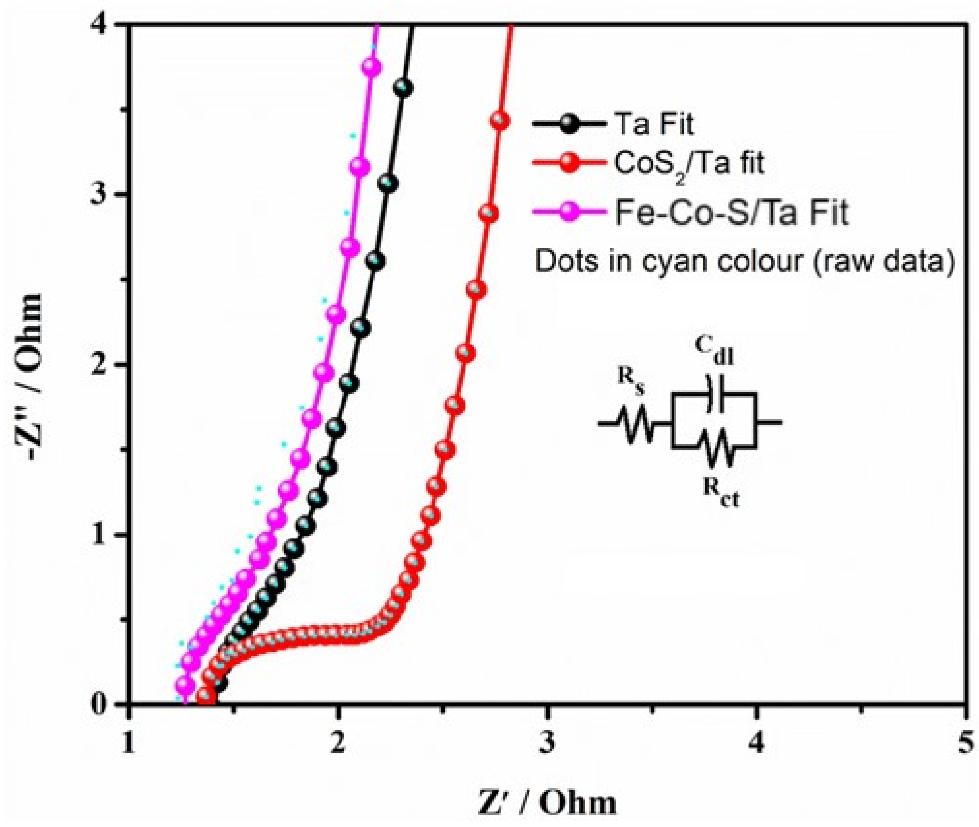

Fig. S14 Fitted EIS plot (dots connected with lines) and the raw data points used for fitting (cyan colour dots)

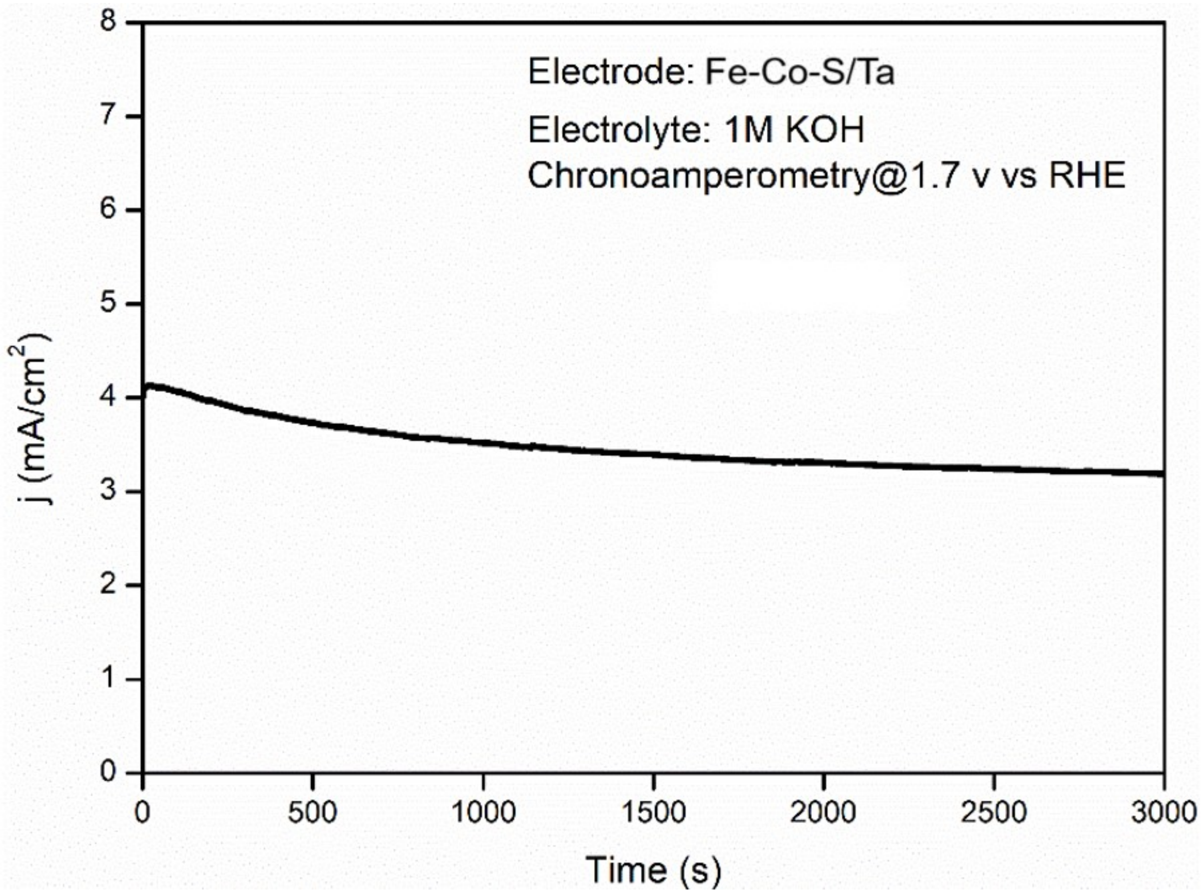

Fig. S15. Current-Time trace of Fe-Co-S/Ta interface measured using 1 M KOH using chronoamperometry at 1.7 V vs RHE
